# Effects of an Integrated ‘Fast Track’ Rehabilitation Service for Multi-Trauma Patients: A Non-Randomized Clinical Trial in the Netherlands

**DOI:** 10.1371/journal.pone.0170047

**Published:** 2017-01-11

**Authors:** Ans I. E. Bouman, Bea Hemmen, Silvia M. A. A. Evers, Henk van de Meent, Ton Ambergen, Pieter E. Vos, Peter R. G. Brink, Henk A. M. Seelen

**Affiliations:** 1 Department of Health Services Research, Faculty of Health, Medicine and Life Sciences, Caphri, School of Public Health and Primary Care, Maastricht University, Maastricht, the Netherlands; 2 Adelante Center of Expertise in Rehabilitation and Audiology, Hoensbroek, the Netherlands; 3 Department of Rehabilitation Medicine, Faculty of Health, Medicine and Life Sciences, Caphri, School of Public Health and Primary Care, Maastricht University, Maastricht, the Netherlands; 4 Department of Rehabilitation Medicine, Maastricht University Medical Center, Maastricht, the Netherlands; 5 Department of Rehabilitation, Radboud University Nijmegen Medical Center, Nijmegen, the Netherlands; 6 Department of Methodology and Statistics, Faculty of Health, Medicine and Life Sciences, Caphri, School of Public Health and Primary Care, Maastricht University, Maastricht, the Netherlands; 7 Department of Neurology, Slingeland Hospital, Doetinchem, the Netherlands; 8 Netwerk Acute Zorg Limburg, Maastricht University Medical Center, the Netherlands; IRCCS E. Medea, ITALY

## Abstract

**Objectives:**

The effects on health related outcomes of a newly-developed rehabilitation program, called ‘supported Fast Track multi-trauma rehabilitation service’ (Fast Track), were evaluated in comparison with conventional trauma rehabilitation service (Care as Usual).

**Methods:**

Prospective, multi-center, non-randomized controlled study. Between 2009 and 2012, 132 adult multi-trauma patients were included: 65 Fast Track and 67 Care as Usual patients with an Injury Severity Score ≥16, complex multiple injuries in several extremities or complex pelvic and/or acetabulum fractures. The Fast Track program involved: *integrated* coordination between trauma surgeon and rehabilitation physician, shorter stay in hospital with faster transfer to a specialized trauma rehabilitation unit, earlier start of multidisciplinary treatment and ‘non-weight bearing’ mobilization. Primary outcomes were functional status (FIM) and quality of life (SF-36) measured through questionnaires at baseline, 3, 6, 9 and 12 months post-trauma. Outcomes were analyzed using a linear mixed-effects regression model.

**Results:**

The FIM scores significantly increased between 0 and 3 months (p<0.001) for both groups showing that they had improved overall, and continued to improve between 3 and 6 months for Fast Track (p = 0.04) and between 3 and 9 months for Care as Usual (p = 0.03). SF-36 scores significantly improved in both groups between 3 and 6 months (Fast Track, p<0.001; Care as Usual, p = 0.01). At 12 months, SF-36 scores were still below (self-reported) baseline measurements of patient health prior to the accident. However, the FIM and SF-36 scores differed little between the groups at any of the measured time points.

**Conclusion:**

Both Fast Track and Care as Usual rehabilitation programs were effective in that multi-trauma patients improved their functional status and quality of life. A faster (maximum) recovery in functional status was observed for Fast Track at 6 months compared to 9 months for Care as Usual. At twelve months follow-up no differential effects between treatment conditions were found.

**Trial Registration:**

ISRCTN68246661

## Introduction

In recent decades, mortality rates in patients with severe injuries have dropped significantly due to advances in medical technology and improved trauma management in the pre-hospital and hospital phases [1–3]. Since a larger number of patients survive their injuries, rehabilitation services have become more important for enhancing a patient’s functional health status, quality of life (QoL) and reintegration into society.

In a literature review covering over 80 studies, Halcomb and colleagues reported that trauma survivors face many problems that affect their functional status, psychological wellbeing, QoL and return to productivity [4], and a multidisciplinary rehabilitation approach seemed to offer the best way to improve trauma patient outcomes, analogous to rehabilitation for stroke patients [5, 6]. In a systematic review, Khan and colleagues aimed to identify studies reporting rehabilitation outcomes for patients with multiple trauma, especially where the approaches were effective. They found some low-quality evidence, according to the Grading of Recommendations Assessment, Development and Evaluation (GRADE) approach, from observational studies to support multidisciplinary intervention in this population [7, 8]. To date, however, there is a lack of comparative studies investigating outcomes between different trauma rehabilitation programs.

As far as we know, this study is the first to compare two different rehabilitation services for multi-trauma patients. In conventional trauma care service (Care as Usual = CAU) in the Netherlands each of the partners have their own more-or-less autonomous treatment perspective, depending on the professional’s individual treatment views and experience [9]. Clinical evidence, gathered by trauma care specialists in the Southern trauma care system, however, suggested that an *integrated* multi-trauma rehabilitation service approach or ‘supported Fast Track multi-trauma rehabilitation service’ (Fast Track = FT) might lead to more effective treatment of multi-trauma patients. This newly-developed approach integrates and co-ordinates the treatment of patients between the trauma surgeon and the rehabilitation physician from an early stage post-trauma. Previous problems, associated with long periods of immobilization after hospital admittance, are being dealt with through an earlier transfer to a specialized rehabilitation clinic. The patients can begin specific training early and benefit from centralized knowledge on multi-trauma rehabilitation. Conceptually, an analogy exists between FT and the concept of ‘stroke units’, which have proven to be (cost-) effective [5, 10]. The effectiveness of FT with regard to functional health status and QoL, however, has not yet been studied. The aim of this study, therefore, is to investigate the effectiveness of the Fast Track rehabilitation program for multi-trauma patients on QoL and other health related measures in comparison to a Care as Usual rehabilitation program over a 12-month follow-up period.

## Methods

### Study design

A prospective, multi-center, non-randomized controlled clinical study compared two multi-trauma rehabilitation services. Patients in the Southern trauma care system of the Netherlands followed the FT treatment and patients in the Eastern trauma care system received the CAU treatment. Because it was not medically or ethically feasible to randomize the acute multi-trauma patients across the two trauma centers, mainly due to geographical distances, a non-randomized study was performed [11]. This study design has a moderate-quality level of evidence according to the GRADE approach [8]. The follow-up period was 12 months with measurements taken at baseline and at 3, 6, 9 and 12 months post-trauma. The patients were included over a period of 34 months from 2009 to 2012. More details of the study design have been published elsewhere [12].

### Participants

All multi-trauma patients admitted to the Accident and Emergency department (A&E) of the participating hospitals were screened for eligibility by the hospital’s team of trauma surgeons and the rehabilitation physician. Multi-trauma was defined as having at least two injuries, of which at least one is life threatening, including a) trauma with an Injury Severity Scale (ISS) ≥16, *OR* b) complex multiple injuries on both lower extremities, *OR* c) a combination of one upper and one lower extremity injury, the latter of which is not allowed load-bearing, *OR* d) complex pelvis/acetabulum fractures [12]. The exclusion criteria were: no hospitalization, less than 18 years old, inadequate Dutch language skills, severe alcohol and/or drug abuse, severe psychiatric/cognitive problems, and no indication for clinical rehabilitation. The rehabilitation indication was determined by the hospital’s rehabilitation physician within a week post-trauma on the basis of a number of factors, including expectation of lasting impairments or handicaps, multiple and complex rehabilitation goals, the patient being trainable, dependence on help from others, and the patient’s motivation to undergo clinical rehabilitation [13]. After the screening procedure at each trauma care system, patients participated in the Fast Track group or in the Care as Usual group.

### Sample size

The sample size was calculated using data from a study by Czyrny and colleagues [14]. They reported an improved motor Functional Independence Measure (FIM) score of 30.2 in a small group of bilateral lower limb multi-trauma patients who had received both hospital and subsequent rehabilitation treatment, at a mean length of stay of 62.8 ±6.0 days. To detect a 15% difference in improvement in this study between FT and CAU group patients at 3 months, assuming a two-sided significance level of 0.05, a power of 80% and a common standard deviation of 9.5 as reported by Czyrny, 71 persons per group were required (142 persons in total) [12].

### Rehabilitation program

#### Fast track

The program for the intervention group patients consisted of an integrated multi-trauma rehabilitation service approach (FT) throughout the trauma care system, featuring:

- Early integrated co-ordination of treatment between the trauma surgeon and the rehabilitation physician at the hospital (within 2 days after hospital admission, with weekly follow-ups)
- Shorter stay in hospital and earlier transfer of multi-trauma patients to a specialized trauma rehabilitation unit (within 5 days after placing the patient on the waiting list of the rehabilitation center)
- Earlier start with specific ‘non-weight bearing’ rehabilitation training at the rehabilitation center
- Earlier start with multidisciplinary treatment, involving a psychologist and a social worker from the first week after admission to the rehabilitation center
- Early individual goal setting
- Structural (monthly) visits from the trauma surgeon to the rehabilitation center for consultation with the rehabilitation team and the patient
- Shorter stay in the trauma rehabilitation unit
- Use of documented treatment protocols.

The duration and intensity of the rehabilitation in the program depended on the type and severity of the patients’ injuries. A detailed implementation plan for the Fast Track program is available from the authors on request.

#### Care as usual

The Care as Usual group patients received the conventional trauma care service, in which the patients are admitted to hospital via A&E. The trauma surgeon will only seek advice from the rehabilitation physician in the early phase of the hospital admission of the trauma patient if there is a spinal cord injury or to discuss whether acute amputation should be performed on severely injured extremities. After surgery, the patients are transferred to the intensive care unit (ICU), where treatment at the request of the intensive care specialist takes place, namely physiotherapy (mobilization) and treatment by a speech therapist (swallowing evaluation and nutrition advice) will start. When trauma patients are stable, they are transferred to the hospital’s nursing ward, where they may stay for several days or weeks. The trauma surgeon, as chief consultant, decides whether or not a rehabilitation physician will be consulted during hospitalization. In effect, involvement of rehabilitation in early stages post-trauma was rather low. In the FT approach, rehabilitation involvement in the early phase was increased, leading to specific treatment protocols and earlier transfer to the rehabilitation center. And therefore, new rehabilitation protocols had to be developed for example on (1) early non weight-bearing mobilization (hydrotherapy) and (2) early involvement of a psychologist and a social worker. Further treatment in the CAU group patients takes place in either a hospital outpatient clinic, a (usually more distant) rehabilitation center, in a nursing home or with a local general practitioner or physiotherapist. Typically, each of these ‘stations’ may have its own more-or-less autonomous treatment perspective, depending on the professional’s individual treatment views and experience. More details of both programs can be found in the trial’s design article [12].

### Outcome measures

It was expected that the Fast Track rehabilitation would improve the health status of the patients more than Care as Usual from baseline to 12 months. The *primary* health-related outcome measures were functional health status and QoL. Functional status was assessed with the FIM, a validated instrument to indicate severity of disability [15, 16]. FIM consists of 18 items, grouped into 2 subscales–Motor and Cognition. QoL was assessed with the Short-form 36 health survey questionnaire (SF-36) [17, 18], measuring health status. All but one of the 36 items (self-reported health transition) generates eight subscales. The *secondary* outcome measure was the anxiety and depression status of the multi-trauma patients. This was assessed with the Hospital Anxiety and Depression Scale (HADS), a validated instrument for screening mood disorders [19]. HADS consists of 14 items, grouped into 2 subscales–Depression and Anxiety.

The outcomes were measured using questionnaires at baseline and at 3, 6, 9 and 12 months post-trauma. The baseline questionnaire also provided, e.g., background characteristics, type of accident, diagnosis, ISS score, Abbreviated Injury Scale (AIS) codes, the number of complications during hospital stay and cognitive functioning measured by the Mini-Mental State Examination (MMSE) [20]. The data were collected through individual interviews by trained research assistants who were not involved in the care of the patients. The questionnaires at 3, 6, 9 and 12 months post-trauma could also be (partly) completed through telephone interviews or by self-reporting through postal questionnaires.

### Statistical analyses

The baseline variables were compared to detect differences between the Fast Track group and the Care as Usual group. Two groups of continuous, symmetrically distributed variables were compared by *t*-tests, and several groups by one-way ANOVA. Mann-Whitney *U*-tests were performed for non normally-distributed data. Categorical variables were compared using Pearson’s Chi-square tests.

#### Primary analyses

The analyses were performed according to the intention-to-treat principle, including all participants with valid data on clinical outcomes, regardless of whether they received the complete intervention or not. The *primary and secondary outcomes* were analyzed in SPSS (version 20.0; SPSS, Inc., Chicago, IL). A linear mixed-effects regression model was used with a two group between variable and four time points (3, 6, 9 and 12 months) were included as a within variable, and also their interaction was included. *The rates of changes over time* within both groups and the difference of the rates of changes over time within both groups was examined, the latter by including the interaction group by time. The mixed-model residuals were checked for normality. All analyses were adjusted for baseline values of the outcome measures, if available, by including the baseline as a covariate. Adjustments were also made for the background characteristics age, sex and educational level, because after major trauma older age, female gender and low education were reported as being detrimental to long-term QoL [21, 22]. Patients were taken random in the model. Two-sided significance tests were used. Mean and standard deviations, adjusted mean differences between the study groups, including 95% confidence intervals (CIs) and p-values are presented.

#### Per-protocol analyses

Per-protocol analyses were also performed. These required that participants in the Fast Track group received the complete integrated FT intervention program, which included a transfer to the specialized trauma unit of the rehabilitation center after their hospital stay. The Care as Usual group consisted of patients who received conventional trauma care service that included inpatient rehabilitation at a rehabilitation center after their hospital stay. Patients needed to have valid data on clinical outcomes.

Ethical approval for the study was obtained from the Medical Ethics Committee of Adelante Rehabilitation Center, Hoensbroek, the Netherlands. Written informed consent was obtained prior to a patient’s participation.

## Results

### Participants

Table 1 shows the baseline characteristics of the 132 participants, 65 Fast Track and 67 Care as Usual group patients. The mean age was 43 years ±16.7. Fast Track group patients had a significantly lower ISS score (22 ±12.8) than Care as Usual group patients (29 ±11.2; p<0.001). More patients had an ISS score <16 in the Fast Track (n = 17) than in the Care as Usual group (n = 3), mostly from complex pelvic fractures (not tabulated). Cross-checking of ISS scoring between the centers (10 cases) was comparable and the difference was not due to a different way of scoring (not tabulated). The differences in the type of injury, that is musculoskeletal injuries versus neuro-trauma/head injuries, were not statistically significant (p = 0.39). Most baseline characteristics were comparable between the groups. Small, but not statistically significant, differences were shown between the groups in baseline values of the outcome measures FIM (p = 0.40), SF-36 (p = 0.17) and HADS (p = 0.86) (Table 1).

**Table 1:** Baseline Characteristics of the Participants.

Characteristic^a^	Sample size (FT/CAU)	Fast Track (n = 65)	Care as Usual (n = 67)	P-value^b^
**Age (y) at injury, mean (SD)**	65/67	44.7 (16.7)	42.0 (16.6)	0.34^1^
Range (y)		18–75	18–73	
**Gender, Male**	65/67	49 (75)	56 (84)	0.24^2^
**Marital status**	64/63			0.54^3^
Married/living together		32 (50)	38 (60)	
Divorced/widowed		12 (19)	5 (8)	
Single		20 (31)	20 (32)	
**Education**	65/65			0.20^3^
Elementary school/lower (professional) education		13 (32)	15 (40)	
Middle (professional) education		16 (40)	16 (42)	
Higher (professional) education		11 (28)	7 (18)	
**Informal care, Yes**	64/65	51 (80)	58 (89)	0.13^2^
**Pre-trauma health disorders, Yes**	63/66	45 (71)	39 (59)	0.14^2^
**Pre-trauma work status, Employed**	62/62	39 (63)	40 (64)	0.85^2^
**Type of accident**	65/64			0.77^3^
Traffic accident		41 (63)	39 (61)	
Fall		15 (23)	15 (23)	
Other		9 (14)	10 (16)	
**Type of injury**	65/67			0.39^3^
Multi-trauma (neuro-trauma and musculoskeletal injuries)		14 (22)	33 (49)	
Musculoskeletal injuries only		48 (74)	28 (42)	
Neuro-trauma		3 (5)	6 (9)	
**ISS, score 0–75, mean (SD)**	64/67	22.1 (12.8)	29.4 (11.2)	<0.001^1^
Range		4–66	4–50	
Median (IQR)		19.5 (12–29)	29 (21–38)	
**Complications during hospital stay, Yes**	61/66	19 (31)	37 (56)	0.01^2^
**MMSE, score 0–30, mean (SD)**^**c**^	58/47	26.6 (4.4)	26.9 (3.4)	0.69^4^
**FIM, score 18–126, mean (SD)**^**d**^	55/60	89.3 (25.0)	93.9 (32.9)	0.40^1^
**SF-36, score 0–100, mean (SD)**^**d**^	37/40	89 (8.8)	86 (12.9)	0.17^1^
**HADS, 0–42, mean (SD)**^**d**^	55/51	11.7 (8.8)	12.0 (8.2)	0.86^1^

CAU, Care as Usual; FIM, Functional Independence Measure; FT, Fast Track; HADS, Hospital Anxiety and Depression Scale; IQR, interquartile range; ISS, Injury Severity Score; MMSE, Mini-Mental State Examination; SD, standard deviation; SF-36, Short Form 36 health survey questionnaire.

^a^ Values are numbers (percentages) unless stated otherwise.

^b^ Significant *p*-value set at 0.05, two-tailed: 1) independent sample *t*-test, 2) Pearson’s Chi-square test, 3) one-way ANOVA, 4) Mann-Whitney U-test.

^c^ Scores of 25 or higher are considered as normal cognitive functioning. A number of patients were not able to perform the test due to injury severity.

^d^ Baseline value of the outcome measure for participants included in the mixed-model analysis. The SF-36 score represents the baseline measurement of the patient’s health in the week *prior* to the accident.

**Fig 1:**
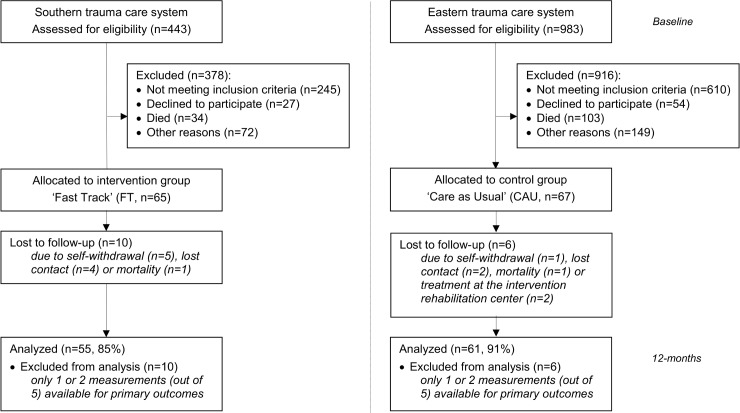
Flow Diagram of the Participants.

The integrated FT intervention program was used throughout the trauma care system and already part of the daily routine, but waiting lists for the rehabilitation center occurred, more physical therapists had to be appointed for the intensive inpatient rehabilitation training and staff at key positions had to be reminded to adhere to FT procedures. All trauma patients were screened for eligibility in both trauma regions. Sixty-five participants were included in the Fast Track program and 67 patients in the Care as Usual program. The original inclusion period of 19 months was, however, extended to 34 months to (nearly) obtain the required number of participants in each study group. The latter was the only study protocol deviation. Of the included Fast Track participants, 40 Fast Track patients (out of 65, 62%) visited the specialized trauma unit and received the complete integrated FT intervention program. Of the included Care as Usual participants, 40 patients (out of 67, 60%) received inpatient care (CAU) at a rehabilitation center (not tabulated). A flow diagram of the participants is shown in Fig 1.

### Outcome measures

The baseline measurement was available for all 132 patients. When patients had more than 2 out of 5 missing questionnaires, they were omitted from the analyses on outcome measures. This was the case for 10 Fast Track group patients (out of 65, 15%) and 6 Care as Usual group patients (out of 67, 9%). The reasons for discarding the patient data were, e.g., self-withdrawal (5 FT, 1 CAU), lost contact (4 FT, 2 CAU) and mortality (1 FT, 1 CAU). Outcome measures were thus available for 116 persons: 55 in the Fast Track (85%) and 61 in the Care as Usual group (91%) (Fig 1). Of those 116 patients, 34 FT (52%) and 39 CAU (59%) completed all 5 questionnaires, 12 FT (19%) and 14 CAU (21%) completed 4 questionnaires, and 9 FT (14%) and 8 CAU patients (12%) completed 3 questionnaires. The outcome values of the missing questionnaires were not substituted as the used mixed-model regression analysis is robust against missing values [23].

#### Primary outcomes

A summary of the results for the primary outcome measures is shown in Table 2. The mixed-model regression analysis was adjusted for small differences in FIM baseline values and for age, sex and education (Table 1). The results showed that few differences in FIM scores could be detected between the two groups at 3, 6, 9 and 12 months post-trauma. Neither were there significant differences in SF-36 scores between the groups at any of the measured time points from 3 to 12 months post-trauma. Because the SF-36 scores at 0 months represent self-reported measurements of the patients’ health status in the week *prior* to the accident, the analysis was only adjusted for age, sex and education. The results of the SF-36 at 12 months showed that QoL had not (yet) reached self-reported pre-trauma baseline levels in both groups. The mean pre-trauma scores were 89 for the FT group (68 at 12 months) and 86 for the CAU group (70 at 12 months) (Tables 1 and 2).

**Table 2:** Mixed-model Analyses over Four Time Points for the Primary and Secondary Outcomes.

		Fast Track (n = 55)			Care as Usual (n = 61)					
Outcome measure^a^	Time point (mo)	Sample size^b^	Mean	SE	Sample size^b^	Mean	SE	Mean difference	95% CI	P-value
***PRIMARY***										
**Functional status**, FIM, score 18–126 ^c^	3	46	115.9	1.73	50	116.3	1.64	-0.38	(-5.12 to 4.36)	0.87
	6	41	118.9	1.48	48	118.0	1.38	0.86	(-3.16 to 4.88)	0.67
	9	43	119.9	1.34	54	119.7	1.24	0.16	(-3.50 to 3.81)	0.93
	12	52	119.0	1.34	57	120.6	1.26	-1.56	(-5.24 to 2.12)	0.40
***PRIMARY***										
**Quality of life**, SF-36, score 0–100	3	48	59	3.1	47	66	2.9	-6.2	(-14.8 to 2.3)	0.15
	6	42	66	2.9	48	72	2.7	-5.9	(-13.9 to 2.0)	0.14
	9	45	69	2.7	54	70	2.5	-0.5	(-8.0 to 6.9)	0.89
	12	51	68	2.9	56	70	2.7	-1.9	(-9.7 to 5.9)	0.64
***SECONDARY***										
**Anxiety and depression status**, HADS, score 0–42	3	49	8.8	1.16	46	9.0	1.14	0.21	(-3.00 to 3.34)	0.90
	6	42	8.2	1.11	46	7.5	1.04	-0.70	(-3.72 to 2.34)	0.65
	9	45	7.3	1.02	54	8.6	0.94	1.38	(-1.38 to 4.14)	0.32
	12	51	7.9	0.99	56	8.9	0.93	1.06	(-1.64 to 3.76)	0.44

CI, confidence interval; FIM, Functional Independence Measure; HADS, Hospital Anxiety and Depression Scale; mo, month; SE, standard error; SF-36, Short Form 36 health survey questionnaire.

^a^ The mixed-model regression analysis over four time points at 3, 6, 9 and 12 months post trauma for FIM and HADS was adjusted for the baseline values and for age, sex and education (Table 1). The analysis for SF-36 was adjusted for age, sex and education. The presented adjusted values at 3, 6, 9 and 12 months and their confidence intervals (CIs) are similar to the unadjusted values (not presented). Positive between-group differences indicate a more favorable score for the Fast Track group.

^b^ Sample sizes may be lower than the group sizes due to a maximum of 2 out of 5 missing questionnaires.

^c^ Underlined score indicates the most-favorable score.

Differences in baseline values between the groups were reported for ISS scores and the number of complications during hospital stay. We therefore added these variables as covariates to the statistical model, but this did not change the results of the primary outcome measures (not tabulated). The type of injury was also exploratory added as covariate, but again, this did not change the results.

The FIM Motor and Cognition subscale analyses showed that the Fast Track group scored significantly better than the Care as Usual group on cognitive functioning at 3 months (not tabulated). The mean difference at 3 months was 1.6 points (FT 33.8 versus CAU 32.3; p = 0.02). The data from the FIM cognition subscale were skewed, but the mixed-model residuals followed a normal distribution and the standard statistical model was applied. From the eight SF-36 subscales, the subscale Physical functioning (10 items) showed significant differences between the groups in favor of the Care as Usual group at 3, 6 and 12 months; the mean differences were, respectively, 18 points (FT 48 versus CAU 66; p = <0.001), 14 points (FT 62 versus CAU 76; p = 0.01) and 11 points (FT 65 versus CAU 76; p = 0.03). The SF-36 subscale Bodily pain (2 items) also showed a significant difference in favor of the Care as Usual group at 12 months; the mean difference was 17 points (FT 68 versus CAU 85; p = <0.001) (not tabulated).

#### Secondary outcomes

Table 2 also shows the results for the secondary outcome HADS. The mixed-model analysis was adjusted for possible differences in HADS baseline values and for age, sex and education. Few differences in HADS scores were found between the groups at 3, 6, 9 and 12 months post-trauma. The Anxiety and Depression subscales showed no significant differences between the groups at any of the measured time points.

#### Rates of changes over time

The results of the mixed-effects regression model showed a significant interaction of group by time. Table 3 shows the rates of changes over time for the primary and secondary outcomes. The mean differences represent the changes in outcomes *within* each study group for the time intervals 0 to 3 months, 3 to 6 months, 6 to 9 months and 9 to 12 months. The interval 0 to 3 months is not available for SF-36, because the baseline values represent the patient’s health *prior* to the accident. For the time interval between 0 and 3 months, the mean adjusted FIM scores (95% CI) for the FT group increase from 88.0 (80.1–95.8) to 115.5 (112.0–119.0) and for CAU from 93.7 (86.2–101.3) to 116.7 (113.4–120.0) (not tabulated). The mean differences are, respectively, 28 and 23 points, as shown in Table 3. The estimated means for the outcome measures in Table 3 (not tabulated) may differ slightly from the means presented in Tables 1 and 2 due to differences in the mixed model used.

**Table 3:** Mixed-model Analyses for the Primary and Secondary Outcomes: Rates of Changes over Time within Both Groups.

	Fast Track (n = 55)				Care as Usual (n = 61)			
Outcome measure^a^^,^^b^	Time interval (mo)	Mean difference	95% CI	P-value	Time interval (mo)	Mean difference	95% CI	P-value
***PRIMARY***								
**Functional status**, FIM, score 18–126 ^c^	0–3	28	(19.8 to 35.3)	<0.001	0–3	23	(15.4 to 30.4)	<0.001
	3–6	2.9	(0.1 to 5.7)	0.04	3–6	1.7	(-1.0 to 4.3)	0.21
	6–9	1.0	(-0.7 to 2.8)	0.23	6–9	1.7	(0.1 to 3.3)	0.03
	9–12	-0.9	(-2.1 to 0.3)	0.15	9–12	0.9	(-0.3 to 2.0)	0.13
***PRIMARY***								
**Quality of life**, SF-36, score 0–100	3–6	6.6	(1.7 to 11.5)	<0.001	3–6	6.3	(1.5 to 11.1)	0.01
	6–9	3.3	(-0.5 to 7.1)	0.08	6–9	-2.1	(-5.6 to 1.5)	0.25
	9–12	-0.9	(-4.5 to 2.7)	0.63	9–12	0.5	(-2.9 to 3.9)	0.78
***SECONDARY***								
**Anxiety and depression status**, HADS, score 0–42	0–3	3.1	(0.7 to 5.5)	0.01	0–3	3.1	(0.6 to 5.7)	0.02
	3–6	0.6	(-1.3 to 2.6)	0.50	3–6	1.5	(-0.4 to 3.4)	0.11
	6–9	0.9	(-0.6 to 2.4)	0.24	6–9	-1.2	(-2.7 to 0.3)	0.12
	9–12	-0.7	(-1.9 to 0.5)	0.28	9–12	-0.3	(-1.5 to 0.9)	0.60

CI, confidence interval; FIM, Functional Independence Measure; HADS, Hospital Anxiety and Depression Scale; mo, month; SF-36, Short Form 36 health survey questionnaire.

^a^ The SF-36 baseline score represent the patient’s pre-trauma health and the interval from 0 to 3 months is therefore not available.

^b^ The mixed-model regression analysis over five time points at 0, 3, 6, 9 and 12 months post trauma for FIM and HADS was adjusted for age, sex and education. For SF-36 the same model was used over four time points from 3 to 12 months. Positive mean differences per time interval indicate a more favorable change in scores within both groups.

^c^ Underlined score indicates the most-favorable score.

The increase in FIM scores within both groups between baseline and 3 months were statistically significant (p<0.001). Between 3 and 6 months only the Fast Track group significantly improved their FIM scores (p = 0.04). A similar improvement in functional status for the Care as Usual group occurred 3 months later (3–9 months, p = 0.03, not tabulated). The rates of changes in FIM between 6–9 and 9–12 months for the Fast Track and between 9–12 months for the Care as Usual group were not statistically significant. The SF-36 showed a significant improvement between 3 and 6 months within both groups (FT, p<0.001 and CAU, p = 0.01) and the HADS between baseline and 3 months (FT, p = 0.01 and CAU, p = 0.02). Table 3 shows that the largest improvements, on average, in functional health status and QoL take place, approximately, up to 3 to 6 months post trauma for patients in both groups.

#### Per-protocol analyses

Per-protocol analyses were carried out for 69 participants (33 FT and 36 CAU). These analyses were based on Fast Track patients receiving inpatient FT rehabilitation at the specialized unit (40 out of 65, 62%) compared to Care as Usual patients receiving inpatient CAU rehabilitation at a rehabilitation center (40 out of 67, 60%). Of those, however, 7 Fast Track group patients and 4 Care as Usual group patients were omitted from the statistical analysis, because they missed more than 2 out of 5 questionnaires. The results of the primary and secondary outcome measures for the smaller group of 69 participants (33 FT and 36 CAU) were similar to the ones described for the overall group analysis of 116 participants (55 FT and 61 CAU, Table 2). Again, few differences were found between the groups at any of the measured time points. The results from the per-protocol analyses are available from the authors on request.

## Discussion

Both the Fast Track and Care as Usual rehabilitation programs were effective in that multi-trauma patients improved their functional status and quality of life. A faster (maximum) recovery in functional status was observed for Fast Track at 6 months compared to 9 months for Care as Usual. Improvements in QoL for both groups were shown up to the first 6 months post-trauma. At twelve months follow-up no differential effects between treatment conditions were found. In both groups, a similar proportion of patients (around 60%) received an inpatient multidisciplinary rehabilitation approach. Measurements from patients were available at several time points, which allowed for advanced statistical analysis, and the drop-out rate was reasonable. No adverse effects were found in this study.

Baseline differences were found between FT and CAU for some variables. However, adjusting the analyses for baseline differences did not affect the results. The FIM cognition subscale showed a positive effect at 3 months on the cognitive status in favor of the Fast Track group. The SF-36 subscales ‘Physical functioning’ and ‘Bodily pain’ showed favorable effects for the Care as Usual group. These results may be explained by the occurrence of relatively more head injuries in the Care as Usual group and more lower-extremities injuries in the Fast Track group. Both types of injuries have been associated with a poor prognosis in patient outcomes [22, 24].

The findings of the intention-to-treat and the per-protocol analyses on the outcome measures at the different time points were similar—no additional more favorable effect of the per-protocol analyses on outcomes was seen for FT than with CAU. A definitive assessment of the merits of the program will be made depending on the effects on (health) care use. The use of all (health) services will be reported in another article, together with a cost-effectiveness and cost-utility analysis.

The present study is to the best of our knowledge the first clinical study, albeit non-randomized, to focus on the effect of early rehabilitation for multi-trauma patients. The original distinguishing features of the FT program did not seem to have contrasted enough with the CAU program and more favorable results for the FT approach may be expected after optimizing procedures. The FT implementation may benefit from an early involvement of the management teams and financing of framework conditions, such as extra beds and appointment of extra therapists at the specialized trauma rehabilitation unit. Regular training of junior doctors in the hospital is necessary as they frequently switch positions. Short lines of communication between medical specialists and the rehabilitation team are needed. The FT program will benefit from appointing a contact person who, with the aid of a set of indicators, ensures that the required actions, such as fast transfer to the specialized unit of the rehabilitation center, are timely.

There were methodological drawbacks. Randomization did not take place, which could have introduced (unknown) confounding factors that were not controlled for. The multi-trauma patient groups may have been too heterogeneous for comparison. In a review by Deeks and colleagues on the evaluation of non-randomized intervention studies, they reported on the complexity of biases in non-randomized studies [25]. The internal validity was considered important and the creation of the intervention groups and the comparability of the groups at the analysis stage were seen as the two most essential domains for evaluating non-randomized studies. Our groups differed on ISS scores and matching did not take place by design as the latter was practically impossible. The results should therefore be interpreted with caution.

The measurement scales were internationally accepted instruments, but ceiling effects for the FIM scale have been reported [26]. In a recent review by Hoffman and colleagues on outcome measures used in major trauma, the SF-36 was most frequently used and the FIM was also often used. However, they concluded that the existing outcome measures did not fully describe the impact of major trauma on function, disability and health. The health outcomes of trauma patients may therefore not be fully understood [27]. The Trauma Outcomes Profile (TOP), a trauma-specific tool, was shown to cover the largest representation of the International Classification of Function, Disability and Health (ICF). The TOP may have potential use in trauma population but requires further validation [28]. The outcome evaluation and understanding of the true health impact of injury may be limited due to the lack of an ICF-based framework [27].

Other factors may have affected the effectiveness of the program. First, the sample size calculation indicated that 71 persons were needed in each group. The intention-to-treat analysis of primary outcomes was based on 55 Fast Track and 61 Care as Usual group patients, which was somewhat lower than anticipated. The data set size upon which the original calculations were based on was, however, small and the total required number may have been overestimated [14]. The power was calculated again (at least 0.80) and the estimated numbers were on the high side. With the use of covariates, the required number may also be lower than the originally calculated 142 persons. Nevertheless, the sample size may have been too small to detect sufficient effects. Second, 60% of the included Fast Track patients (33 out of 55) received the specific ‘non weight-bearing’ rehabilitation training and multidisciplinary treatment. This may have diluted possible additional beneficial effects. It is still possible, however, that the FT program may not have added enough to the already existing high-standard services in the Dutch health care system. About the same proportion of FT and CAU patients included in the statistical analysis received inpatient rehabilitation. Of the CAU group patients, 59% (36 out of 61) also benefited from a multidisciplinary rehabilitation program at the rehabilitation center.

A review by Khan and colleagues on multidisciplinary rehabilitation in patients with multiple trauma highlighted the lack of high-quality studies for effective multidisciplinary rehabilitation in survivors of multiple trauma. Only low-quality evidence from observational studies supported multidisciplinary intervention in this population [7]. The present study is the first clinical trial that compared two multidisciplinary rehabilitation programs for multi-trauma patients (with moderate-quality evidence according to GRADE) [8]. Both programs showed positive effects on functional status and QoL. Other reviews investigating multidisciplinary rehabilitation programs for specific diagnostic groups (stroke, after hip or knee joint replacement, persons with multiple sclerosis, and acquired brain injury) all suggest that multidisciplinary rehabilitation is an effective intervention [5, 29–31].

Heterogeneity in major trauma populations, study designs (only observational), use of different outcome measures and rehabilitation specifications (intensity, duration or content mostly not described at all) complicate comparison of outcome results. There are, however, common findings that are compatible with the results in this study. Outcomes at 12 months showed that the SF-36 scores for both study groups were still below self-reported pre-trauma levels and below normative data for the Dutch population [32]. In another Dutch study (22% of major trauma patients went to a rehabilitation center), considerable levels of disability and impaired QoL were also reported 12–18 months after hospitalization [22]. Major trauma patients who survive injury and do not always return to pre-injury (functional) health status were described in several other studies [33–40]. A first attempt to compare QoL outcomes in major trauma patients from regional trauma registries in Hong Kong and the Victorian State Trauma Registry in Australia showed no difference in 6-month and 12-month functional outcomes between the jurisdictions [41].

Recent developments within rehabilitation show an increased interest in interventions aiming at early mobilization and improved collaboration between different medical specialties [42]. These are key features of the Fast Track program. The medical importance of applying the FT concept is, in relation to collaboration in health care, that the treatments in the trauma departments and the rehabilitation departments in the hospitals and rehabilitation centres are better coordinated. This means that the collaboration between not only the doctors of different specialisms but also the paramedics involved in the rehabilitation treatment is improved. This, in turn, leads to concentration of expertise, i.e. knowledge of and experience with the rehabilitation of multi-trauma patients.

The principle behind FT may be generalised to other settings and other patient groups. Following this study, the FT concept was successfully implemented in other trauma regions in the Netherlands using a well-described implementation plan (available from the authors on request). One example of this generalisability to other patient groups is the application of the FT concept to treating patients after a lower limb amputation. Integrated collaboration between vascular surgeons and rehabilitation teams in the Southern trauma care region made possible early mobilisation of these patients. Moreover, the present research can serve as a model for evaluating other (related) care chains. Policy makers at national level can use this research as an example because it is demonstrably the first study in which a treatment for multi-trauma patients is clearly set out in protocols and is systematically a) set up in a complex interaction area of clinical and paramedical disciplines, b) tested and studied for (cost) effectiveness and c) reported.

Based on the results of this study, the following recommendations can be made:

- To further optimize primarily the clinical and outpatient rehabilitation treatment in FT in terms of scope and duration
- To identify indicators to facilitate early identification of multi-trauma patients who have not recovered as much of their functional health and quality of life at 12 months as originally expected (i.e. ‘poor performers’)
- To develop and evaluate training modules for multi-trauma patients who have not recovered as much of their functional health and quality of life at 12 months as originally expected (i.e. ‘poor performers’)
- To give further form to the rehabilitation treatment at the earliest stage (in ICU) in order to optimise the condition of the patient as far as possible and to prevent complications arising from inactivity.

## Conclusions

Both the Fast Track and Care as Usual rehabilitation programs were effective in that multi-trauma patients improved their functional status and QoL. A faster (maximum) recovery in functional status was observed for Fast Track patients at 6 months compared to 9 months for Care as Usual patients. At twelve months follow-up no differential effects between treatment conditions were found. The FT program may prove to be more advantageous for patients’ outcomes, but a detailed process evaluation alongside a replication of this study is needed to screen adherence to protocol procedures and to solve bottlenecks as soon as possible. Randomized stratified trials to investigate the effectiveness of new rehabilitation concepts are preferred in view of the wide range of injuries that multi-trauma patients experience, but may be difficult to conduct for ethical and practical reasons.

## Supporting Information

S1 Table Baseline Data from the Participants (n = 132). (SAV)

S2 Table Data from the Measurements at 0, 3, 6, 9 and 12 Months Post Trauma for the Mixed-model Analyses (n = 116). (SAV)

S1 File TREND checklist. (PDF)

S2 File Study Protocol Kosar et al 2009. (PDF)

S3 File Clinical Trial Protocol submitted to the Medical Ethics Committee. (PDF)

## Abbreviations

A&E: Accident and Emergency department
AIS: Abbreviated Injury Scale
CI: Confidence Interval
CAU: Care as Usual (conventional trauma care service)
FIM: Functional Independence Measure
HADS: Hospital Anxiety and Depression Scale
ICF: International Classification of Function, Disability and Health
ICU: Intensive Care Unit
IQR: Interquartile Range
ISS: Injury Severity Score
MMSE: Mini-Mental State Examination
QoL: Quality of Life
SF-36: Short-form 36 health survey questionnaire
FT: Fast Track (supported Fast Track multi-trauma rehabilitation service)
SPSS: Statistical Package for the Social Sciences
TOP: Trauma Outcomes Profile
