# Supplementary material for: Effects of an Integrated ‘Fast Track’ Rehabilitation Service for Multi-Trauma Patients: A Non-Randomized Clinical Trial in the Netherlands
Source: PLoS One. 2017 Jan 11;12(1):e0170047. doi: 10.1371/journal.pone.0170047 (PMC5226800; doi:10.1371/journal.pone.0170047)
Supplement: S3 File — (PDF) [file pone.0170047.s005.pdf]

DEFINITIEF

Algemene gegevens / General Information

Programma / Programme

DoelmatigheidsOnderzoek: deelprogramma Effecten & Kosten

Subsidieronde / Subsidy round

E&K - round 08 - grant application

Projecttitel / Project title

Supported Fast track multi-Trauma Rehabilitation Service (SFTRS)

Aanvrager / Applicant

Dr. H.A.M. Seelen
Functie / Position: | *Opleiding / Education:*
Studierichting / Subject:
T: 045 5282221 | *F:* 045 5282227 | *E:* h.seelen@srl.nl
Stichting Revalidatie Limburg

Postbus 88
6430 AB HOENSBROEK

Projectleden / Project members

Dr. H.A.M. Seelen (Projectleider en penvoerder)
Functie / Position: Coordinator Onderzoek & programmaleider SRL | *Opleiding / Education:*
Studierichting / Subject:
T: 045 5282221 | *F:* 045 5282227 | *E:* h.seelen@srl.nl
Stichting Revalidatie Limburg

Postbus 88
6430 AB HOENSBROEK
Nederland

Drs. T.S. de Wit (Bestuurlijk verantwoordelijke)
Functie / Position: Algemeen directeur SRL | *Opleiding / Education:*
Studierichting / Subject:
T: 045 5282001 | *F:* | *E:*
Stichting Revalidatie Limburg

Postbus 88
6430 AB HOENSBROEK
Nederland

Prof. dr. P.R.G. Brink (Mede aanvrager)
Functie / Position: Hoogleraar Traumatologie | *Opleiding / Education:*
Studierichting / Subject:

T: 043 3875491 | *F:* | *E:* p.brink@surgery.azm.nl
Academisch Ziekenhuis Maastricht
Traumacentrum Limburg

Postbus 5800
6202 AZ MAASTRICHT
Nederland

Dr. mr. S.M.A.A. Evers (Uitvoerder)
Functie / Position: Senior Onderzoeker HTA | *Opleiding / Education:*
Studierichting / Subject:
T: 043 3881602 | *F:* | *E:* s.evers@beoz.unimaas.nl
Universiteit Maastricht
Faculteit der Gezondheidswetenschappen
Capaciteitsgroep Beleid & Economie in de Organisatie van de Zorg - BEOZ
Postbus 616
6200 MD MAASTRICHT
Nederland

Dr. B. Hemmen (Mede projectleider)
Functie / Position: Revalidatiearts | *Opleiding / Education:*
Studierichting / Subject:
T: 045 5282320 | *F:* | *E:* b.hemmen@srl.nl
Stichting Revalidatie Limburg

Postbus 88
6430 AB HOENSBROEK
Nederland

Dr. H. van der Linde (Uitvoerder)
Functie / Position: Revalidatiearts | *Opleiding / Education:*
Studierichting / Subject:
T: 024 3658768 | *F:* | *E:* h.vdlinde@planet.nl
Sint Maartenskliniek
Research & Ontwikkeling

Postbus 9011
6500 GM NIJMEGEN
Nederland

Prof. dr. J.L. Severens (Projectadviseur)
Functie / Position: Hoogleraar Health Technology Assessment | *Opleiding / Education:*
Studierichting / Subject:
T: 043 3882907 | *F:* | *E:* H.Severens@beoz.unimaas.nl
Universiteit Maastricht

Faculteit der Gezondheidswetenschappen
Capaciteitsgroep Beleid & Economie in de Organisatie van de Zorg - BEOZ
Postbus 616
6200 MD MAASTRICHT
Nederland

Vacature (Uitvoerder)
Functie / Position: HTA specialist | *Opleiding / Education:*
Studierichting / Subject:
T: 045 5282221 | *F:* | *E:*
Universiteit Maastricht
Faculteit der Gezondheidswetenschappen
Capaciteitsgroep Beleid & Economie in de Organisatie van de Zorg - BEOZ
Postbus 616
6200 MD MAASTRICHT
Nederland

Vacatures (Uitvoerder)
Functie / Position: Health scientist + trial nurses | *Opleiding / Education:*
Studierichting / Subject:
T: 045 5282221 | *F:* | *E:*
Stichting Revalidatie Limburg

Postbus 88
6430 AB HOENSBROEK
Nederland

Prof. dr. A.B. van Vugt (Projectadviseur)
Functie / Position: Hoogleraar Traumatologie | *Opleiding / Education:*
Studierichting / Subject:
T: 024 3610455 | *F:* | *E:* TRO@medzaken.umcn.nl
Universitair Medisch Centrum St. Radboud
Traumacentrum

Geert Grooteplein-Zuid 10
6525 GA NIJMEGEN
Nederland

Samenwerking / Collaboration

Universitair Medisch Centrum St. Radboud
Traumacentrum

Geert Grooteplein-Zuid 10
6525 GA NIJMEGEN

Maaslandziekenhuis

Postbus 5500
6130 MB SITTARD

Atrium Medisch Centrum

Postbus 4446
6401 CX HEERLEN

Sint Maartenskliniek

Postbus 9011
6500 GM NIJMEGEN

Academisch Ziekenhuis Maastricht
Revalidatie

P Debyelaan 25
6229 HX MAASTRICHT

Universitair Medisch Centrum St. Radboud

Postbus 9101
6500 HB NIJMEGEN

Sint Maartenskliniek
Research & Ontwikkeling

Postbus 9011
6500 GM NIJMEGEN

Academisch Ziekenhuis Maastricht
Traumacentrum Limburg

Postbus 5800
6202 AZ MAASTRICHT

Stichting Revalidatie Limburg

Postbus 88
6430 AB HOENSBROEK

Universiteit Maastricht
Faculty of Health, Medicine and Life Sciences
Capaciteitsgroep Beleid & Economie in de Organisatie van de Zorg - BEOZ
Postbus 616
6200 MD MAASTRICHT

Projectgegevens / Project information

Datum indienen (via ProjectNet) / Date of application

12-02-2007 11:54

Aandachtsgebieden / Focus

Themes:

Organisatie van zorg;

Themes HTA methodology:

Outcome measures ; Evaluation research of aspects of organisation; Health outcomes research/Implementation;

Projecttype / Project type

Onderzoeksproject

Samenvatting / Summary

NL:
Doel: Het hoofddoel is de effectiviteit, de kosten en de kosteneffectiviteit te onderzoeken van een geïntegreerd vroege revalidatie-interventie zorgketen (SFTRS) voor multi-trauma patiënten. De SFTRS wordt gecontrasteerd met conventionele multi-trauma zorg. Centraal in de SFTRS staan 1) betrokkenheid van revalidatiearts in een zeer vroeg stadium post-trauma; 2) een snellere transfer van multi-trauma patiënten vanuit het ziekenhuis naar een gespecialiseerde traumarevalidatie-afdeling; 3) een vroegere start van specifieke, multidisciplinaire revalidatiebehandeling met a) gewichtsondersteunende trainingsmodules, psychologische en sociale counselling b) het vroeg stellen van individuele revalidatiedoelen, c) een geïntegreerde coördinatie van de behandeling tussen trauma chirurg, revalidatiearts en revalidatiebehandelteam; 4) korter verblijf in ziekenhuis en revalidatiecentrum; 5) goed gedocumenteerde behandelprotocollen.

Vraagstelling: Welke van 2 zorgketens, i.e. conventionele multi-trauma zorg of SFTRS is het meest (kosten-)effectief?

Studie opzet: In een prospective, multi-centre, non-randomised clinical trial met een duur van de follow-up van 12 maanden zullen 164 (2x 82) multi-trauma patiënten deelnemen. Een groep van patiënten zal volgens het SFTRS regime behandeld worden, terwijl de tweede groep de conventionele multi-trauma zorg ontvangt.

Studiepopulatie: Multi-trauma patiënten met een ISS>=16 zullen geïncludeerd worden.

Interventie: Een multidisciplinaire revalidatiebehandeling volgens SFTRS wordt vergeleken met conventionele multi-trauma zorg.

Uitkomstmaten: De primaire uitkomstmaten zijn 'kwaliteit van leven' en 'functionele

gezondheidstoestand' . Secundaire uitkomstmaten zijn: 'angst en depressie', 'cognitief functioneren' en de 'mate waarin de gestelde ADL behandeldoelstellingen bereikt zijn'. Ook zullen 'kosten' geregistreerd worden.

Sample size / data analyse: Om een verschil in FIM score van 4.5 (gebaseerd op data van patiënten met bilaterale multiple beentraumata, gerapporteerd door Czyrny et al. 1998) te detecteren, zijn 71 personen nodig. Bij 15% 'loss to follow-up' zijn 82 personen per groep (164 voor beide groepen) nodig voor de studie. Verschillen in uitkomsten tussen beide groepen zullen geanalyseerd worden via meerdere MANCOVA's.

Economische evaluatie: Een 'cost-utility' analyse wordt uitgevoerd vanuit een maatschappelijk standpunt.

Tijdpad: Mnd 1-3: voorbereiding, mnd 4-21: inclusie, mnd 22-33: follow-up, mnd 34-36: definitieve analyses en rapportage.

EN:
Objective: The main objective is to examine the effectiveness, the costs and the cost-effectiveness of an integrated 'fast track' rehabilitation service for multi-trauma patients (SFTRS) involving dedicated early rehab intervention programs. The SFTRS is contrasted with conventional multi-trauma care'. In the SFTRS the rehabilitation physician is
routinely involved in a very early stage post-trauma, facilitating early start of multidisciplinary rehab treatment involving a) early non-weight bearing physical therapy, psychological and social counselling, b) early transfer to a centralised, specialised trauma rehab unit equipped with facilities for early training programs, c) early individual rehab goal setting, d) close co-operation and exchange of views and experiences between the trauma surgeon, the rehabilitation physician and the rehab team. SFTRS also features shorter lengths of stay and well documented treatment protocols.

Research question: Which of 2 services, i.e. 'Conventional multi-Trauma Care Service' or 'Supported Fast track multi-Trauma Rehabilitation Service' (SFTRS), is most (cost-)effective?

Design: In a prospective, multi-centre, non-randomised clinical trial 164 (2x 82) mult-trauma patients will participate. The duration of follow-up is 12 months. One group of patients will follow the SFTRS treatment, whereas the second group will receive conventional multi-trauma care.

Population: Multi-trauma patients with an ISS>=16 will be included in the study.

Intervention: A multidisciplinary rehabilitation treatment according to SFTRS will be compared to conventional multi-trauma care.

Outcome measures: Primary outcome measures are 'quality of life' and 'functional health status'. Secondary outcome measures are 'anxiety and depression', 'cognitive functioning' and 'extent to which individual ADL treatment goals are met'. Also costs will be assessed.

Sample size / analysis: To detect a difference in FIM score of 4.5 (based on data in bilateral lower limb multi-trauma patients by Czyrny et al., 1998) between both services, 71 persons are needed per group. At 15% loss to follow-up, the required group size is 82. For both groups 164 persons are required. Differences in outcome measures between both groups will be analysed using multiple MANCOVA's.

Economic evaluation: A cost-utility analysis, will be performed from a societal perspective.

Time schedule: Month 1-3: preparation, month 4-21: inclusion, month 22-33: follow-up, month 34-36: final analyses and reporting.

Trefwoorden / Keywords

multiple trauma, rehabilitation, clinical trial, functional status, cost-effectiveness

Inhoud / Content

Probleemstelling / Problem definition

- Health care problem:

Annually +/- 99.000 people are admitted to hospital after an accident. 880.000 people visit the accident & emergency department (A&E) after an accident (Stg. Consument & Veiligheid, 2005). These accidents lead to considerable societal costs. Direct medical costs are estimated at 1 billion euro annually, i.e. 3-4% of the total Dutch health care budget. Production losses due to acute trauma are estimated at 4 billion euro, thus widely surpassing costs associated with chronic illness like cardiovascular diseases and cancer (Mulder, 2002; Weseman, 2000; Beeck, 1997). Based on the 1997 bill "Met Zorg Verbonden" (Ministries of VWS and Internal Affairs) and the bill "Beleidsvisie Traumazorg" in 1999, 10 trauma centres were allocated nation-wide (van Vree et al., 2001). Medical care for trauma victims is a combined responsibility of hospitals, ambulance services, trauma centres, rehabilitation clinics, and GHOR (geneeskundige hulpverlening bij ongevallen en rampen). This co-operation is called trauma care chain (TCC). The azM is the trauma centre in Limburg (Hammacher, 2004). In conventional multi-trauma care service (CTCS) each of the partners has its own more or less autonomous treatment perspective, depending on the professional's individual treatment views and experience. Clinical evidence, however, suggests that an integrated multi-trauma rehabilitation service approach or 'Supported Fast track multi-Trauma Rehabilitation Service' (SFTRS), featuring:
1) shorter stay in hospital and earlier transfer of multi-trauma patients to a specialised trauma rehabilitation unit
2) an earlier start of both specific 'non-weight bearing' rehab training and multidisciplinary treatment
3) early individual goal setting
4) an integrated co-ordination of treatment between trauma surgeon and rehabilitation physician
5) shorter stay in trauma rehab unit

may be more (cost-)effective. Conceptually, an analogy can be drawn between the SFTRS approach and the concept of 'stroke units' which have proven to be cost-effective (EDISSE project, ZONMW; Exel, 2003; Launois, 2004). Costs for using a hospital bed are higher than for a bed in a rehab centre (Oostenbrink, 2004).

- Disease/condition:

Multi-trauma is defined as having at least 2 or more traumatic injuries, of which at least one is life threatening (Zelle, 2005a). Several tools for rating trauma severity have been designed (Cooper, 2004). The Injury Severity Score (ISS) (Baker, 1974) is used most. Major causes of multi-trauma are traffic accidents, accidents at work, (extreme) sports, falls, blasts, etc. (Erli, 2000). Several studies report that the legs (incl. pelvis) are most frequently injured in multi-trauma (Holbrook, 2001a,b).

- (Sub)group of patients:

Multi-trauma occurs more often in males and in younger adults (Erli, 2000; www.prismant.nl, 2004). Many patients are at an age where they have a paid job.

- Usual care in the Netherlands for this (sub)group of patients:

In conventional multi-trauma care service (CTCS) patients are admitted to hospital via the A&E. After

possible surgery, they are transferred to the IC-unit, followed by the general surgery ward, where the patient may stay for several days/weeks. The trauma surgeon seeks the advice of the rehabilitation physician, if necessary. Ensuing treatment takes place in a hospital's outpatient clinic, in a (more distant) rehab centre, in a nursing home or with a local general practitioner or physiotherapist. Van Vree, (2001) reported that, typically, each of the 'stations' in the CTCS may have its own more or less autonomous treatment perspective, depending on the professional's individual treatment views and experience.

- Disciplines involved in the usual care in the Netherlands for this (sub)group of patients:
General surgeons, orthopaedic surgeons, anaesthetists, rehabilitation physicians, physiotherapists, occupational therapists, nurses

- Disciplines in the usual care and their participation in this proposal:
General surgeons, orthopaedic surgeons, anaesthetists, rehabilitation physicians, physiotherapists, occupational therapists, nurses, psychologists, social workers are involved in CTCS and/or SFTRS.

- Motivation for intervention and the effectiveness of the intervention:
Ample evidence is available confirming that each of the separate treatment elements within the TCC is effective. However, ambiguity as to general outcome exists. Dimopoulou, (2004) report that the majority of multi-trauma patients still suffer from considerable levels of disability and impaired quality of life 1 year post-trauma. Van der Sluis, (1995) reported moderate to poor functional outcome in 26% of patients after 2 years. It is likely that earlier trãnsfer of multi-trauma patients from hospital to a specialised trauma rehab unit, leading to a faster discharge from rehabilitation, will lead to better functioning of patients, both somatically and psycho-socially. The proposed study aims at investigating which of 2 services (CTCS or SFTRS) is more (cost-)effective.

Relevantie / Relevance

- Motivation for chosen theme
The study fits theme 8: organisation of care. It compares the (cost-)effectiveness of 2 multi-trauma services, i.e. conventional multi-trauma care service (CTCS) and SFTRS, which differ as to organisation. The effects of the underlying therapy / care components have been established in numerous studies. However, as reported in the systematic review, contrasts in cost-effectiveness between multi-trauma rehab services have not been studied. The SFTRS features:
a) shorter lengths of stay (in hospital and in the specialised multi-trauma rehab unit) through improved logistics within the TCC
b) closer co-operation between trauma surgeon, rehab physician and the rehab team
c) earlier start with specific comprehensive rehab
training modules early post-trauma
d) earlier integrated multidisciplinary treatment, and rehab goal setting
leading to:
* optimisation of treatment
* reduction of secondary complications
* reduction of function loss associated with prolonged bed rest (e.g. muscle atrophy, endurance loss, contractures)
* achievement of an optimal level of functioning, participation and quality of life

- Contribution of results
The study will yield results on the efficiency of an adapted care service for multi-trauma patients (SFTRS) featuring earlier (and condensed) involvement of specialised rehab treatment. Results will show whether improved SFTRS logistics, combined with shorter stays in hospital and rehab clinic and specialised early rehab training modules are more (cost-)effective, relative to CTCS. Quality of life and functional recovery not only depend on injury severity, but also on prevention of secondary

complications, psychological/social factors (Erli, 2000; Zelle, 2005b), and patient's cognitive status (Fernandez, 2001). The early integrated rehab approach in SFTRS focuses on these issues specifically.

- Similar studies
In stroke rehab the development of stroke units proved to be cost-effective (EDISSE project, ZONMW; Exel, 2003; Launois, 2004). In the EDISSE study, stroke patients in 3 experimental stroke service settings were compared with patients receiving usual stroke care in a 6 months follow-up. Results showed that integrating services for acute stroke lead to organisational improvements, higher efficiency and better outcome (e.g. reduced hospital length of stay, less inappropriate hospital days, more satisfied patients). The EDISSE study also provided criteria for optimising the quality of stroke services. The SFTRS is similar in its aims (i.e. higher efficiency, better patient's outcome), its approach (i.e. integrating services for multi-trauma patients, especially in the early phases post-trauma) and its design (i.e. prospective, non-randomised clinical trial).

- Recent reports
The reports below call for further optimisation of multi-trauma care, supporting the SFTRS concept: .
- Government bill "Met Zorg Verbonden", Ministries of VWS and Internal Affairs (1997)
- Government bill "Beleidsvisie Traumazorg" (1999), VWS
- Vree van, 2001. Beleidsvisie traumazorg. Eerste inventarisatie stand van zaken, CVZ
- Gouma, 2002. RGO advies onderzoek traumazorg
- Hammacher, 2004. Traumatologie in perspectief: Beleidsplan Nederlandse Vereniging voor Traumatologie

- Incidence/prevalence of targeted (sub)population
A nation-wide database for trauma patients is not yet fully operational. Annually, 99.000 people are admitted to hospital after an accident. 880.000 trauma victims visit the accident & emergency department (Stg. Consument & Veiligheid, 2005). In Limburg the incidence of multi-trauma patients with an ISS >=16 is about 170/year (Trauma centre database azM, 2005-2006). At the UMC St. Radboud trauma centre this number is 260/year (see letters in annex).

- Potential effects on health
Earlier start of specific rehab treatment after multi-trauma includes a.o. non-weight bearing physical training, multidisciplinary treatment of the patient, personalised goal setting and early start of psychological and social counselling. The SFTRS approach will lead to less secondary complications associated with bed rest, which would negatively influence recovery and quality of life. Early personalised goal setting and early treatment of depression are known to positively affect outcome. SFTRS will lead to faster reintegration into society. Early return to work and active support from the multidisciplinary rehab team will lead to a more stable social network, and the patient becoming less reliant on professional care in the long term.

- Potential effects on costs
SFTRS will reduce the length of stay of multi-trauma patients in a hospital. Earlier rehab treatment in a specialised rehab unit will also reduce the length of stay in the rehab clinic, thus reducing costs of hospital/clinic consumption. At this moment it is not possible to make a precise calculation of these savings. Since earlier discharge also means that patients take part in society and work earlier, costs related to production losses and patient&family costs are expected to be lower.

Kennisoverdracht, implementatie, bestendiging / Knowledge transfer, implementation, consolidation

- aim
Aim for knowledge transfer and/or implementation is to inform health care professionals in the field of

multi-trauma care throughout the Netherlands about the results of the study and to train them on how to implement the SFTRS approach into the trauma care chain (TCC). An implementation strategy will be developed in the last phase of the project, depending on the results of the study.

- Results
Results will be disseminated through publications in peer-reviewed journals, presentations at (international) congresses, symposia, the Dutch Societies for Traumatology (NVT) and Rehabilitation (VRA), and the 'werkgroep Traumarevalidatie (WTR)'. Furthermore, the SFTRS will lead to 'spin-off' regarding:
a) collaboration among TCC members as to courses, training of staff and exchange of knowledge; .
b) further development of treatment protocols for the whole TCC;
c) further elaboration of the role of traumatology in the medical curricula (CHIVO);
d) intensifying research collaboration and multi-centered TCC research projects;
If proven (cost-)effective, SFTRS will be implemented in other trauma centres / TCC's throughout the Netherlands. An implementation plan as to this purpose will be drawn in the last phase of the project, depending of the project results.

- Organisations to take notice of the study results
Professionals involved in multi-trauma patient care in hospitals and rehabilitation centres in the Netherlands (specialistenverenigingen NVT, WTR, Nederlands vereniging voor Heelkunde (NVvH), Nederlandse Orthopaedische Vereniging (NOV), Federatie Traumatologie i.o.) and abroad (international trauma / rehab associations), TCC specialists: Trauma surgeons, A&E specialists, registrars in rehabilitation medicine, physiotherapists, occupational therapists, psychologists, social workers, university hospitals, regional hospitals, rehabilitation centres/depts, trauma centres, ministry of VWS, Raad voor GezondheidszorgOnderzoek, College van Zorgverzekeraars, Revalidatie Nederland, VRA, GG&GD's.

- Organisations who should integrate the results in their activities:
Professionals involved in multi-trauma patient care in hospitals and rehabilitation centres in the Netherlands (specialistenverenigingen NVT, WTR, NVvH, NOV, Federatie Traumatologie i.o.) and abroad (international trauma / rehab associations), TCC specialists: Trauma surgeons, A&E specialists, registrars in rehabilitation medicine, physiotherapists, occupational therapists, psychologists, social workers, university hospitals, regional hospitals, rehabilitation centres/depts, trauma centres.

Doelstelling / Objective

Main objective
The main objective of this study is to examine the effectiveness, costs and cost-effectiveness of an integrated care service for multi-trauma patients involving:
a) faster transfer of patients in an earlier post-trauma phase from the traumatology dept. to a specialised rehabilitation clinic
b) earlier involvement of registrar in rehabilitation medicine in the treatment of the patient
c) earlier start of multidisciplinary rehabilitation training, tailored to individual patient's need, including a.o. earlier partial weight bearing training, individual goal setting, prevention of complications associated with prolonged inactivity (e.g. muscle athrophy, reduced mobility, reduced cardiovascular endurance) and psychological and social counselling
d) earlier discharge of patients from the rehabilitation clinic, leading to faster and better participation in society
e) improvement of patient care through improvement and centralisation of expertise regarding rehabilitation of multi-trauma patients
f) improvement of communication among (para-)medical staff within the TCC, shorter lines of communication by intensifying contacts between trauma surgeons and registrars in rehabilitation

medicine
This approach is called 'Supported Fast track multi-Trauma Rehabilitation Service' or SFTRS.

The general research question is:

Which of 2 rehabilitation services, i.e. 'Conventional multi-Trauma Care Service' (CTCS) or 'Supported Fast track multi-Trauma Rehabilitation Service' (SFTRS), is most (cost-)effective from a societal point of view?

Sub-questions are:

What are the effects of the SFTRS on generic quality of life in multi-trauma patients as compared to the CTCS?

What are the effects of the SFTRS on functional health status as compared to the CTCS?

What are the costs to health care and to society of the SFTRS as compared to the CTCS?

What is the cost-effectiveness of the SFTRS as compared to the CTCS?

Plan van aanpak / Strategy

Clinical study
- preliminary studies by applicants

Henk A.M. Seelen, PhD, has performed (and led) several experimental and clinical studies into the effects of major trauma (e.g. traumatic spinal cord injury, limb amputation and stroke) on patients' performance and outcome of training, for which he has obtained several grants (ZONMW / NWO, EU, Industry). Furthermore, he is leading a project on optimisation of integrated post-rehab care in high cervical spinal cord injured persons requiring 24-hours ventilation.

Peter G.R. Brink, PhD, professor of Traumatology, has conducted numerous trauma-related clinical studies, focussing on intensive care medicine and trauma surgery. He is strongly involved in the organisation of trauma care in the Netherlands and especially in Limburg.

Bena Hemmen, MD, PhD, has conducted several studies on treatment outcome in stroke and orthopaedic trauma. Together with S. Evers and H. Seelen she investigated the (cost-)effectiveness of computer-controlled prostheses in leg amputees. She is starting a large multi-centre study into functional assessment and early prognostics in traumatic amputees, in collaboration with H. Seelen, H. van der Linde and Prof. J. Geertzen, MD, PhD (UMC Groningen). Together with Prof. Brink and Dr. Seelen she currently is performing a study on early prognostics in multi-trauma patients. The latter project is made possible because of the close co-operation between the trauma surgeons and rehabilitation physicians from the azM, the hospitals in Heerlen and Sittard and the Hoensbroeck Rehabilitation Centre. Publications on the results of the project are in progress.

Silvia M.A.A. Evers, MA, PhD, has specific experience with trial based health technology assessment studies in a.o. stroke and pain. She is (and has been) involved as a HTA supervisor in several ZONMW studies.

Arie B. van Vugt, PhD, professor of Traumatology, is specialised in effectiveness and quality of trauma care research in severely injured patients. As to projects related to the current proposal: he has led many research projects on treatment and outcome in patients who have suffered accident injuries. He

has specific expertise in the area of hip, pelvis and heel bone fractures.

Harmen van der Linde, MD, PhD, has led several projects related to outcome of treatment in lower limb amputation and prosthetics. He is strongly involved in the development of guidelines for prosthetics prescription in the Netherlands.

- design

A prospective, multi-centre, non-randomised clinical trial will be performed. Two multi-trauma rehabilitation services will be contrasted, i.e. 'Conventional Trauma Care Service' (CTCS) and 'Supported Fast track multi-Trauma Rehabilitation Service' (SFTRS). Duration of follow-up is 12 months, with measurements taken at 3, 6 and 9 months post-injury.

As soon as possible post-trauma (T0) the following data will be recorded:
- General patient characteristics
- Diagnosis
- ISS score (Baker et al., 1974)
- Date and time of trauma
- Trauma treatment
- time in hospital
- Glasgow Coma Scale (Teasdale et al., 1974) upon arrival at the A&E
- Complications: description, number and extent (Santini et al., 2004)

Upon arrival at the rehab centre additional data are collected:
- Individual rehabilitation treatment aims (COPM) (Carswell et al., 2004)
- Pre-trauma psycho-social status (Erli et al., 2000)
- Pre-trauma employment status (Zelle et al., 2005b)
- Depression (HADS) (Spinhoven et al., 1997)

During the rehabilitation phase the following data are recorded:
- Length of stay (in hospital, rehab clinic, out-patient rehab)
- Therapy contents
- Workers' compensation status (Zelle et al. 2005b)

Outcome measures (see below) are collected at baseline, 3, 6, 9 and 12 months post-trauma, i.e. T0, T1, T2, T3 and T4.

- study population

Multi-trauma patients admitted to one of the accident & emergency departments (A&E) of the participating hospitals are included. Multi-trauma is defined as having at least 2 or more injuries of which at least 1 is life threatening.

Inclusion criteria:
- Age >=18 yrs
- multi-trauma
- Injury Severity Scale (ISS)score >=16
- Hospitalisation after A&E admission
- Rehabilitation indication, i.e. lasting impairments or handicaps are expected
- Adequate Dutch language skills

Exclusion criteria:
- Alcohol and/or drug abuse
- Severe psychiatric problems

- interventions
SFTRS
The Supported Fast track multi Trauma Rehabilitation Service (SFTRS) is provided in the province of Limburg by the combined efforts of the Trauma Centre Limburg, the Department of Amputation & Traumatology at the Hoensbroeck Rehabilitation Centre and the Trauma Surgery and Rehabilitation departments of the hospitals in Maastricht, Sittard and Heerlen.

The contrasts with conventional multi-trauma care service (CTCS) are:
a) The rehabilitation physician from the rehabilitation centre is routinely involved at a very early stage post-trauma. This allows an early start for multidisciplinary rehab treatment.
b) Early transfer (within five days after being added to the waiting list from the rehabilitation centre) to a centralized, specialized trauma rehabilitation unit equipped with facilities for early training programs.
c) Early individual rehabilitation goal setting.
d) Close co-operation and exchange of views and experiences between the trauma surgeon and the rehabilitation team by, for example, monthly clinical sessions and individual patient visits by the trauma surgeon in the first weeks after discharge.
e) Well-documented treatment protocols for multi-trauma patients for both the hospital and rehabilitation centre phases.

Three phases can be identified in the treatment of multi-trauma patients:
1 Early rehabilitation phase
2 Stage II rehabilitation phase
3 Discharge or post-discharge phase

Phase 1: Early rehabilitation phase
In the early rehabilitation phase, the patient is not allowed to mobilize weight bearing. Consequently, the physiotherapist is concerned with maintaining joint mobility, muscle strength, sitting balance, condition and training transfers as well as treatments with non-weight-bearing conditions such as hydrotherapy and non-weight-bearing gait training. There are 10 sessions per week of 30 minutes each. In addition, fitness, gymnastics, table tennis, swimming, bowling, hand bike, wheelchair training, and archery are given. There are 2-3 sessions per week for each treatment modality of 60 minutes each.

The occupational therapist advises on bed posture, mattress types, aids for independent daily self-care, wheelchair-dependency training and meaningful activities that can be performed while bedridden. In addition, the wheelchair accessibility and wheelchair friendliness of the patient's home are studied. If necessary, written and oral advice on temporary and long-term adaptations to the home is given and support is given and the patient is helped to apply for financial support so that the patient can return home as soon as possible. At first, this would be for a day or two at the weekend, supervised by an occupational therapist, but would later become permanent. With regard to work, the patient's job is analysed and the patient's workplace is visited. There are 4 sessions per week of 30 minutes each.

The social worker and the psychologist will see every multi trauma patient within the first week after admission. The social worker helps the patient to return home by dealing with the family and offering advice and support to the patient on financial matters, transport facilities. The social worker also contacts the employer and company doctor to look into the possibility of reintegrating the patient into their present job.

The psychologist will examine the patient with regard to such things as mood disorders, posttraumatic

stress syndrome (PTSS), acceptance problems and cognitive problems. The latter requires extensive neuropsychological testing. In addition, individual and group psychological counselling and specialized treatment for PTSS are given. If necessary, the rehabilitation specialist can refer the patient to a Consultant Psychiatrist, Consultant Neurologist, Consultant Medicine, Consultant Rheumatologist and/or Consultant Urologist, who come to the rehabilitation centre.

Phase 2: Stage II rehabilitation phase
In the Stage II rehabilitation phase, new treatment aims are added by the physiotherapist. These might include a gradual individual weight bearing scheme, coordination training and functional training. There are 7 therapy sessions per week of 30 minutes. In addition, fitness, gymnastics, table tennis, swimming, rowing, cycling and archery are given. This is offered in 2-4 sessions per week for each treatment modality of 60 minutes each.

The occupational therapist continues with the treatment goals as mentioned for Phase I and trains the patient to perform household tasks, hobbies, etc in a home-like environment. There are three sessions per week of 30 minutes each. In addition, group therapies such as occupational therapy and recreational therapy are given 2-4 times per week each.

The social worker and the psychologist continue the work mentioned in Phase I, depending on the individual needs of each patient.

Phase 3: (Post) discharge phase
In the discharge phase, the patient is prepared for living at home and is referred to local physiotherapists, specialized sport clubs and mental health care professionals.

CTCS
Conventional multi-trauma care service (CTCS) is provided in several centres. Multi-trauma patients are admitted to hospital via the A&E department. After possible surgery, they are transferred to the IC-unit, followed by the hospital's nursing ward, where the patient may stay for several days or weeks. The trauma surgeon, as chief consultant, decides whether or not a rehabilitation physician will be consulted during hospitalization. Next, ensuing treatment may take place in the hospital's outpatient clinic, in a rehabilitation centre, in a nursing home or with a local GP or physiotherapist. Van Vree and co-authors (2001) reported that, typically, each of the CTCS "stages" might have its own more-or-less autonomous treatment perspective, depending on the professional's individual treatment views and experience.

The effectiveness of multi-trauma rehabilitation interventions and its constituting elements has been established in numerous studies. Even most recently, Holtslag (2007), in his PhD research, investigated the long term outcome after major trauma. Furthermore, effectiveness of multi-trauma care has been established by others (e.g. Cameron et al., 2004), as reported in the systematic review (see below). In the group of van Vugt, Frankema and colleagues (2002) have investigated the quality of extamural and intramural trauma care.

- outcome parameters

Primary outcome measures:
- Generic quality of life: SF-36 (vd Zee et al., 1993)
- Functional health status: Functional Independence Measure (FIM) (Kidd et al., 1995)

Secondary outcome measures:
- Extent to which individual ADL treatment goals are met: COPM
- Anxiety and depression: HADS

- Cognitive functioning: MMSE

Next to that costs will be assessed using the PRODISQ, a cost questionnaire and hospital databases.

- Argumentation for the chosen measures.

In the current study the primary outcome measures are quality of life and functional health status. In several studies it was found that in multi-trauma patients quality of life and functional recovery do not solely depend on injury severity and complications (Holbrook et al., 2001), but also on psychological and social factors (e.g. Richmond et al. 1998; Erli, 2000; Halcomb et al. 2005; Zelle, 2005b) as well as the patient's cognitive status (Fernandez, 2001). The functional independence measure (FIM) is widely used in assessing functional health status in different groups of patients. Baldry-Currens (2000) recommended using the FIM in assessing trauma outcome, the FIM correlating high with measures of injury severity and demonstrating clinical and statistical significance. Similarly, Hetherington et al (1995) reported that in rehabilitation services,the FIM is a useful, practical and simple methodology, providing a measure for assessing the original disability, its progress and residual limitations. The National Trauma Data Bank collects data on trauma centre performance throughout the US. As to functional outcome assessment in trauma patients FIM data are used (Nirula et al., 2006).

At an international and interdisciplinary consensus conference in 1999 about the assessment and application of quality of life (QoL) measures after multiple trauma, experts clinicians and methodologists agreed on the SF-36 as generic tools for QoL assessment across all trauma patients (Neugebauer et al., 2002). In the proposed study both generic QoL and utilities will be derived using the SF-36. An overall utility score for population based QoL can be obtained, which facilitates comparisons with other interventions, i.e. the social tariff of the SF-36 (Brazier et al. 1992, 2002).

The Canadian Occupational Performance Measure (COPM) is an individualised client-oriented measure to assess the evolution of self-perception of skills in patients across time (McColl et al., 2000; Kinebanian et al. 2006). The COPM was, for example, used by Trombly et al (2002) to investigate the association between participation in goal-specific outpatient occupational therapy and improvement in self-identified goals in adults with acquired brain injury. In our study the COPM will be used to assess the extent to which individual treatment aims of the multi-trauma patient, set during rehabilitation, are met.

The Hospital Anxiety and Depression Scale (HADS) gives clinically meaningful results as a psychological screening tool, in clinical group comparisons and in correlational studies with several aspects of disease and quality of life. It is sensitive to changes both during the course of diseases and in response to psychotherapeutic and psychopharmacological intervention. Finally, HADS scores predict psychosocial and possibly also physical outcome (Hermann, 1997). The HADS has been used by Kempen et al. (2003) to investigate the effect of depressive symptoms on the recovery of activities of daily living after fall-related injuries to the extremities in older persons. As stated before, anxiety and depression a.o. may influence therapy outcome in multi-trauma rehabilitation. Therefore, in the present study the HADS is used to assess this aspect.

The Mini-Mental State Examination (MMSE) is a test that briefly surveys global mental status in a wide range of cognitive domains (Jackson et al. 2007; Folstein, 1975). Jackson et al (2007) used the MMSE in trauma survivors without intracranial hemorrhage. Their findings corroborated earlier research stating that these patients display persistent cognitive impairment associated with functional defects, poor quality of life, and an inability to return to work (Jackson et al., 2007). In our study the MMSE will be used similarly, i.e. to assess global cognitive functioning of multi-trauma patients.

- Data collection (see also flowchart)

T0: (upon arrival at the A&E)

- General patient characteristics

- Diagnosis
- ISS score
- Date and time of trauma
- Glasgow Coma Scale (GCS)

As soon as possible post-trauma the following data are collected
- Trauma treatment
- Complications: description, number and extent
- Time in hospital

Upon admission in the rehab centre additional data are collected:
- Individual rehabilitation treatment aims
- Pre-trauma psycho-social status
- Pre-trauma employment status
- Credibility/expectancy questionnaire (CEQ) (Devilly & Borkovec, 2000)
- SF-36, FIM, COPM, HADS, MMSE, cost questionnaire

During the rehabilitation phase the following data are recorded:
- Length of stay (in hospital, rehab clinic, out-patient rehab)
- Therapy contents
- Workers' compensation status

T1: SF-36, FIM, COPM, HADS, MMSE and cost questionnaire will be administered individually at 3 months post-trauma

T2: SF-36, FIM, COPM, HADS, MMSE and cost questionnaire will be administered individually at 6 months post-trauma

T3: SF-36, FIM, COPM, HADS, MMSE and cost questionnaire will be administered individually at 9 months post-trauma

T4: SF-36, FIM, COPM, HADS, MMSE and cost questionnaire will be administered individually at 12 months post-trauma

- Sample size calculation

To date, no exact data on differences in quality of life outcome between different treatment services for multi-trauma patients are available. Czyrny et al. (1998) report an improvement in motor FIM score of 30.2 in a small group of bilateral lower limb multi trauma patients having received both hospital and subsequent rehabilitation treatment, at a mean length of stay of 62.8 (+/-6.0) days. In order to detect a difference of 15% of such improvement in FIM score between SFTRS and CTCS at T1 (assuming a two-sided significance level of 0.05, a power of 80%, and a common standard deviation of 9.5 as reported by Czyrny et al. (1998)), 71 persons per group are needed. Taking into account a 15% loss to follow-up, the required sample size is 82 persons per group (164 persons in total). Cave: This number may be an overestimation, as the Czyrny study involved only a low number of subjects, thus augmenting the sd.

- feasibility of recruitment

Most recent influx number of multi-trauma patients with an ISS>=16 in the Limburg trauma centre region was approx. 170 annually and 260/year for the UMC St. Radboud adherence(see letters in annex). These numbers are expected to stay stable in the coming years. Inclusion time in this study is 18 months, resulting in approx. 250 and 390 patients in the respective regions, totalling 640 for this study.

In 2005 38.2% of all multi-trauma patients in the South Limburg area were admitted to Hoenbroeck Rehabilitation Centre. Referal was approx. evenly distributed across the year, equivalent to 5+ patients per month. For the Nijmegen region 8+ patients per month are expected to be referred to the rehab department. Patient influx time for the proposed project is 18 months. Based on these data it is expected that at least 234 multi-trauma patients can enter this study, (n=13 per month) which is well over the number needed.

- data analysis and presentation

In non-randomised comparative studies, variations in case mix between centres can influence the interpretation of outcome data (Davenport et al. 1996). Therefore, for each of the data sets collected at T1 through T4, differences in outcome variable between the 2 services will be tested using multiple MANCOVA's, entering various indicators of case mix as co-variates, i.e. age, gender, ISS, number of complications, pre-trauma psycho-social status. Results will presented in peer reviewed (inter)national papers and congresses.

When patients drop out of the study, the reason for their withdrawal will be recorded. Drop-outs may bias the treatment effect evaluations. Therefore, the following regime will be applied:
- Missing T4 measurement: 'last-observation-carried-forward' principle will be applied.
- Missing T3 or T2 measurement: linear interpolation of data using data from adjacent time points (e.g. T1 and T4)for imputation.
- Missing T0 or T1 measurement or more than 2 missing measurements: discarding of patient data and influx of additional patient in order to meet n=82 per group.

- treatment credibility and expectancy

In studies comparing the effectiveness of different treatment regimes, differences in treatment credibility and expectancy may influence the outcome. In the proposed study the credibility/expectancy questionnaire (CEQ) (Devilly & Borkovec, 2000) will be administered directly following the explanation of the study's rationale to patients, i.e. after informed consent has been obtained.

- economic evaluation

General considerations

For the economic evaluation the main research question is:
From the viewpoint of the society is another organisation of professional care service for trauma patients (i.e. SFTRS) compared to CTCS preferable in terms of costs, effects and utilities?
Based on this main research question several sub-questions are relevant:
1) What are the costs of SFTRS compared to CTCS preferable in terms of costs, effects and utilities?
2) What are the extra effects (measured in quality of life, utilities, and saving by reducing inpatient hospital admissions of multi-trauma patient) of SFTRS compared to CTCS preferable in terms of costs, effects and utilities?
We hypothesise that SFTRS is associated with a reduction in health care and patient costs, and an improvement in quality of life, compared to CTCS. We expect SFTRS to be cost-effective from a societal perspective. Assessments of the quality of life and costs will take place at T1 through T4.

Cost-analysis

In the cost identification, the following costs are considered:
- Health care costs: cost of the intervention program and other health care resources both by the patient and the caregiver.

• Patient and family costs: informal care, paid domestic help, transportation, over the counter medication, and other out-of-pocket expenses.
• Production losses: absenteeism, presenteeism (loss of productivity while at work), and compensation mechanisms for both the patient and the caregiver, if relevant.
Measurement of volumes:
• Hours spent on the intervention program will be recorded on a pre-structured form by the acting health care professionals.
• All other health care costs and patient & family costs will be recorded in a cost questionnaire
• Production losses will be measured using the patient modules of the PRODISQ (Koopmanschap et al., 2004, 2005)
The PRODISQ will be used together with the costs questionnaire, every 3 months at baseline and T1 through T4.
For the valuation of health care costs and patient & family costs, an update of the Dutch manual for costing in economic evaluations (Oostenbrink et al. 2004) will be used. For care for which no costs-guidelines are available estimations of the costs will be made, based on the real costs and/or on population based estimates from literature. Valuation of production losses will be based on a modification of the friction cost method which has been developed in the ZonMW project "Measuring & valuing productivity costs in HTA".

Patient outcome analysis
Both generic Quality of life (QoL) and utilities are derived from the SF-36. An overall utility score for population based QoL can be obtained, which facilitates comparisons with other interventions, i.e. the social tariff of the SF-36 (Brazier 1992, 2002).
The primary outcome measure for the cost-effectiveness analysis will be FIM. The primary outcomes measure for the cost-utility measure will be utilities based on the SF-36 social tariff.
The time horizon is 12 months. Ratios will be determined, based on incremental costs and effects of SFTRS compared to CTCS. The cost-effectiveness ratio will be stated in terms of costs per improvement on the FIM. The cost-utility ratio will focus on the net cost per QALY gained. Bootstrap re-sampling techniques (Briggs et al., 1997; O'Hagan, 2003) are used to explore cost-effectiveness uncertainty.
Sensitivity analyses will be performed for the costs that turn out to have the largest impact on the differences in total costs between SFTRS and CTCS. In these analyses both the variance in volumes and prices will be considered. The range over which uncertain factors are thought to vary will be assessed by calculating a minimum and maximum (mean value of costs minus or plus the SD).

- Systematic review

Search terms:

Population: Adult multiple trauma population

Intervention: Multidisciplinary care, rehabilitation

Outcome measure: Quality of life, health status, functional status, length of stay, costs

Methodological filters: Systematic review, RCT, follow-up design

The search string was as follows: Multiple trauma /rehabilitation AND (Rehabilitation OR Multidisciplinary care team OR Functional status OR Quality of life OR Health status OR Treatment outcomes OR

Outcome assessment OR Length of stay OR Costs OR Cost analysis OR Review OR Trial OR Follow-up) NOT (Drugs OR Case report OR Mental Health OR Psychia* OR Brain injury OR head injury OR Animals).
Please note that the term 'Trial' includes studies using other methodological designs than RCT only, according to the MeSH thesaurus.
Only for the Cinahl database the '/rehabilitation' term was omitted from the search string.

Databases:
Medline (1980 – present), Cinahl (1982 – 2006), Cochrane Database of Systematic Reviews, Database of Abstracts of Reviews of Effects, Cochrane Central Register of Controlled Trials, Cochrane Database of Methodology Reviews, Cochrane Methodology Register, Health Technology Assessment Database, NHS Economic Evaluation Database.

Number of manuscripts retrieved:
After having performed the above mentioned search strategy, 281 manuscripts were retrieved: Medline: 89; Cinahl: 186; Cochrane 6.

Selection procedure:
Assessment of these manuscripts resulted in 17 relevant studies. Included were: systematic reviews, trials reporting group treatments and care outcome, including functional outcome, health status, quality of life and multidisciplinary approaches. Not included were studies on specific Accident & Emergency room (A&E) and ICU protocols, A&E and ICU diagnostics, pre-hospital life support, nutritional programmes, metabolic assessments, ventilation-related problems.

Validity assessment:
Two reviewers independently assessed all studies for relevance and quality.

Results:
The total number of selected papers was 17, i.e. 4 (systematic) reviews, 0 RCT's, and 12 papers on clinical trials / follow-up studies, 1 paper reporting a costs study.

Summary and conclusion:
Eastwood (1999) reviewed 23 reports on standardised measures of functional status in rehabilitation. Such measures can be used in determining outcome of interventions, managing rehabilitation services, providing empirical data for formal accreditation of care centres and guiding reimbursement of services, based on the patient's specific needs and severity of impairment. The author concluded that there is a lack of agreement on appropriate tools for functional assessment, in contrast to the urgent need to better understand service efficacy based on outcome measures and accountability in rehabilitation.
Elliott (1999) reviewed 31 papers reporting measures used for patient outcomes assessment in adult ICU care. Apart from mortality rates, functional status, health status and quality of life measures were assessed. It was concluded that, although in the majority of survivors health status and activity levels seem to be similar at follow-up time relative to pre-trauma time, methodological flaws as to control over exclusion, heterogeneity of groups and losses to follow-up seriously hampered comparisons of findings. A strong recommendation for consensus on reporting patients' characteristics data as well as outcome measures to be used was given.
The review by Cameron et al. (2004), including 9 reports, aimed at examining the (cost-) effectiveness of specialised multidisciplinary inpatient rehabilitation supervised by a geriatrician or rehabilitation physician compared with usual (orthopaedic) care for older patients with proximal femoral fracture. All main outcome measures tended to be better for patients receiving co-ordinated inpatient rehabilitation. However, statistically, no differences between groups was found, due to heterogeneity in length of stay

and cost data. None of the studies reported quality of life measures. It was concluded that, although further evidence is necessary, there is some rationale to adopt multidisciplinary inpatient rehabilitation. Furthermore, it was concluded that future trials involving specialised inpatient rehabilitation should aim at a) standardising outcome measures (especially regarding functional status); b) clearly recording patient characteristics, including cognitive status; c) assessing (cost-) effectiveness of multidisciplinary rehabilitation integrally, rather than attempting to evaluate its components.
In the (non-systematic) review by Halcomb et al. (2005) 89 studies related to outcome after severe trauma were assessed. Inadequate support by current trauma systems; referral to rehabilitation facility being affected by a.o. provider preferences; long-term loss of productivity; a high incidence of psychological problems; negative effects of alcohol and drug abuse on outcome and positive effects of social support on recovery after severe trauma are reported. It is recommended that research into trauma rehabilitation interventions aiming at patients' individual needs and individual treatment goals is performed, taking into account the sustainability and (cost-) effective follow-up of such interventions.
Van der Sluis et al. (1995), in a retrospective medical records study, assessed the functional outcome in multiple trauma patients (ISS >=16) (n=723). Age, gender, type of accident, AIS/ISS, discharge destination, length of hospital stay and functional outcome (Glasgow Outcome Scale) were retrieved. Off all patients 25.7% died. Half of the survivors were discharged home and 29% were transferred to a rehabilitation centre. Although the functional outcome deteriorated linearly with increasing AIS/ISS, the final functional result was good. Two years post-injury, 68% had mild or no disabilities, 19% were moderately and 7% severely disabled.
Holbrook et al. (1998, 1999, 2001), in a prospective study, investigated the effects of major trauma on functional outcome and quality of well-being. They found that major complications, occurring in 10.1% of all in-hospital patients (n=820), encompassing foremost pulmonary, gastrointestinal, musculoskeletal problems and infections, led to significant lower quality of life levels at discharge as well as at 6, 12 and 18 months post-discharge. High functional limitation levels were reported in multiple trauma patients (n=826) at 6 months follow-up. At 12 and 18 months follow-up (n=806 and n=780 resp.) approx. 80% of all patients still scored well below healthy norm values as to quality of well-being. Depression, serious extremity injury and length of stay were significant predictors of quality of well-being at 6, 12 and 18 months post-discharge. A negative association between functional outcome and extremity injury was found, in contrast to chest or abdominal injury.
Czyrny et al. (1998) performed a retrospective study (n=33) to assess functional outcome of acute in-hospital rehabilitation in patients with multiple limb trauma. They reported significant increases in functional health status (FIM) after comprehensive intensive early inpatient rehabilitation, mean FIM motor scores differences reaching 28.7 between admission and discharge ($p<0.05$) after an average rehabilitation period of 27 days. The authors mention a.o. the small group size and the lack of a follow-up data set as limitations to their study.
Richmond et al. (1998) investigated predictors of disability in persons after non-neurological trauma (n=109) using questionnaires and medical record data. They found that extremity injuries, high levels of posttraumatic psychological distress and educational level are risk factors for severe disability at 3 months post-discharge (odds ratios 2.9, 2.9 and 3.4 resp.).
The study by Fern et al. (1998) compared resource utilisation and long-term outcome in a group of patients having multiple extremity injuries (n=54) and a control group of patients with other major (non-neurological) injuries (n=18) at a level 1 trauma centre. Results showed that average length of stay was 92% longer and resource usage doubled in the first group. After discharge the first group experienced greater long-term disability and higher productivity losses. The authors conclude that the trauma patients with multiple extremity injuries are a distinct group with special needs, that hospital resources for this group are underestimated and that new instruments to predict health status and resource utilisation are necessary.
Miller et al. (2000) investigated functional outcome in severely injured patients staying more than 3 weeks in an intensive care unit (ICU) (n=115). Approx. 47% of patients were discharged to a

rehabilitation centre with a mean FIM score of 52. After rehab discharge (+/- 48 days) mean FIM score was 86 and improved to 101 at 3 month follow-up. For the rehab group, no clear differences in mean FIM related to age were found.
Erli et al. (2000) investigated predictors of quality of life after multiple trauma (n=173). Results showed that quality of life not only dependent on injury severity, but also on ventilation time, age and psychosocial factors. Rehabilitation highly correlated with long-term quality of life. Shortening prolonged in-patient time also significantly improved quality of life in multiple trauma patients.
Dimopoulou et al. (2004) evaluated health-related quality of life and disability in multiple trauma patients (n=87) up to 1 year post-ICU. Results indicate that, next to somatic complaints, emotional well-being was affected. Approx. 72% of patients experienced problems with daily activities, 47% reported an inability to work whereas 59% felt moderately to severely disabled.
Vles et al (2005) studied the prevalence and determinants of disability and return to work after severe trauma. In the group of survivors (n=196) problems with mobility (34%), self-care (15%), daily activities (51%), pain & discomfort (58%), anxiety & depression (37%) and cognitive ability (57%) were reported. Quality of life scores were well below normative values. They concluded that a quarter of trauma patients of working age were unable to return to work and more than half of the trauma patient group experienced problems in their daily life.
The study of Holtslag et al. (2007) aimed at assessing long-term functional health status in severely injured patients and comparing results to normative data (n=335). Furthermore, relations between functional health status and patient characteristics were assessed. Most problems reported related to work, ambulation, housekeeping, recreation and alertness. Deviation from norm data seemed inversely related to age. Important predictors of psychosocial and physical functioning were age, co-morbidity and injury type.
Häusler et al. (2006) investigated the direct costs and consequential costs of 63 major trauma survivors with an ISS>13. Disability is the most important factor determining trauma costs. 35% of all major trauma patients are permanently disabled and cost in excess of 8.7 times as much as those patients (65%) who recover and are rehabilitated. On average, 2/3 of trauma costs are a result of production loss and other consequential costs. Costs like primary hospital treatment costs represent a minor fraction of the total costs. The authors conclude that it is essential that medical and political decision-makers adopt a comprehensive socio-economic view of trauma costs.

In conclusion, most clinical studies compared single treatment outcome. Only few studies comparing outcome of different treatment regimes in multiple trauma care, either experimental or control regimes, were reported. No RCT on treatment effects in multiple trauma patients was found. Although many studies report the multidisciplinary character of the problems multiple trauma patients encounter, in none of the reviewed studies the multidisciplinary rehabilitation program was described in detail. One study suggests that reducing length of stay may positively influence quality of life. Both methodology and measures used in outcome measurement in multiple trauma studies differ considerably. In many cases reliable data on variation in group data are lacking due to either small group sizes or non-reporting. Several reviews point out the necessity of standardising outcome measures as well as assessing (cost-) effectiveness of multidisciplinary rehabilitation.

- time schedule
Month 1-3: preparation
Month 4-21: inclusion
Month 22-33: completion of follow-up
Month 34-36: final statistical analyses and reporting

Patient influx numbers are described on p.16 (feasibility of recruitment).

A HTA methodology study (title: "Measurement of costs in economic evaluations studying organisation of care: back to ABC")is part of this application and has been submitted separately.

Expertise, voorgaande activiteiten en producten / Expertise, prior activities and products

Peter R.G. Brink is a consultant in general surgery, trauma surgery and intensive care medicine. He holds a PhD and is professor of general surgery-trauma surgery at the Faculty of Medicine at the University of Maastricht. In addition, he is head of the Accident & Emergency Department of the University Hospital Maastricht as well as being head of the Limburg Trauma centre. He is also an extra-mural lecturer at the Catholic University of Leuven, Belgium. He is involved in organizing trauma care in the Netherlands at local, regional and national level. He has been the driving force behind the development of care for trauma patients in the southern Netherlands - not only in clinical work but also in education and research. He holds many international positions in the field of trauma surgery, including Chairman of the Board of the ATLS as well as being an ATLS course director and member of the board of the European Trauma Society. He was a member of the editorial board of the Dutch Journal of Traumatology as well as the Dutch Journal of General Surgery. Prof. Brink (co-)authored over 75 papers.

Arie van Vugt, PhD, is professor of Traumatology, deputy head of the Surgery dept., head of the Accident & Emergency dept. at the UMC St. Radboud in Nijmegen and medical co-ordinator of the Trauma Centrum Oost. He holds numerous international positions in the field of trauma surgery. He is an instructor at many courses in the field of trauma surgery a.o. AO, ATLS, PHPLS and DSTC. He is co-editor of the European Journal of Emergency Surgery & Intensive Care and board member of the British Journal of Surgery, the European Journal of Trauma as well as the Nederlands Tijdschrift voor Geneeskunde. His current lines of research encompass quality and (cost-)effectiveness of trauma care and routine CT-examination in severely injured patients. He was promotor at six PhD theses. Prof. van Vugt (co-)authored over 110 papers.

Bena Hemmen, PhD, is Consultant Rehabilitation and head of the Orthopaedic Rehabilitation dept. at the Hoensbroeck Rehabilitation Centre. She has extensive international experience in general surgery, orthopaedics and rehabilitation. Her research areas are 'Functional added-value of advanced prosthetic care' and 'Rehabilitation innovation for multi-trauma patients'. She is a member of the Dutch Society of Rehabilitation, Dutch Society of Traumatology and the International Society for Prosthetics & Orthotics. Dr. Hemmen (co-)authored 40 papers and congress presentations.

Henk Seelen, PhD, is a movement scientist and scientific research co-ordinator at SRL in Hoensbroek. He is also program leader of the Trauma Rehabilitation research line in SRL. His research focuses on 'Motor control in spinal cord injury and stroke', 'Advanced prosthetics' and 'Trauma rehabilitation'. He is member of the Research Group of the Dutch Flemish Spinal Cord Injury Society, of the Dutch Society of Rehabilitation and of several international scientific societies. He is co-promotor at several PhD theses. Dr. Seelen is (co-)author of over 50 papers and over 80 congress presentations.

Hans Severens, PhD, is professor of Medical Technology Assessment at Maastricht University and co-head of the Department of Clinical Epidemiology and MTA of the University Hospital Maastricht. His main research interests are related to the execution of both empirical and modeling studies regarding the cost-effectiveness of health technologies. In this respects he worked on the validity and reliability of cost measurement and productivity costs and the statistics of incremental cost-effectiveness. Besides this, he shows an interest in the efficiency of implementation strategies. He (co-)authored over 100 papers.

Silvia Evers, PhD, is a senior HTA researcher at the Maastricht University. She wrote several

international publications on economic evaluations on several subjects, a.o. on costing methods. She is an assistant professor at the department of Health Organisation Policy and Economics. A number of projects in which she was co-applicant were refunded mainly on international comparison (OECD Ageing Related Disease project, and the project Cross-national transferability of cost-effectiveness data) and on transferral of organizational care. She is a.o. a member of the Cochrane Health Economics Methods Group and the Mental Health Economics Europe.

Harmen vd Linde, PhD, is Consultant Rehabilitation at the UMC St. Radboud and SMK, Nijmegen. His research interest is in Prosthetics and multitrauma. He is strongly involved in the development of P&O guidelines. He is chairman of the Dutch board of the International Society for Prosthetics & Orthotics, charman of the committee for medical aids of the VRA, and member of the scientific committee of the VRA. He (co-)authored numerous papers on teh above mentioned subjects.

Publicaties / Publications

- Haagh WA, van Pampus EC, van Zutphen SW, BRINK PR. 2006. [Coagulation disorders in patients with trauma to the skull and brain: a frequent and potentially fatal combination] NTvG 150(46):2530-5
- BRINK P, Verbruggen J. 2006. ATLS in Europe. Osteosynth Trauma Care 14:84-9
- Emans PJ, van Zutphen S, BRINK PRG. 2006. Posterieure toegang bij posteromediale tibiapalteaufractuur. NTvT 1:13-19
- van Helden S, Cals J , Kessels F, BRINK P, Dinant G, Geusens P. 2006. Risk of new clinical fractures within 2 years following a fracture. Osteopor Int 17:348-54
- Wieland A, Dekkers G, BRINK P. 2005. Open wedge osteotomy for malunited extra-articular distal radius fractures with plate osteosynthesis without bone grafting. Eur J Traum 31:148-54
- Creusen EJA, BRINK PRG. 2005. De operatie volgens Sauve-Kapandjii. NTvT 13(4):103-8
- Jagers op Akkerhuis M, vd Heijden M, BRINK PRG. 2002. Hyaluronidase versus survival excision of ganglia: a prospective, randomised clinical trial. J Hand Surg [Br] 27(3):256-8.
- Vd Heijden MWJ, Willems PC, BRINK PRG. 2001. Mitek tenodese en vroege functionele nabehandeling bij achillespeesrupturen. Ned Tijdschr Heelk 10(2):38-43
- BRINK PRG. 2000. ATLS Nederland, vief jaar later. Ned Tijdschr Heelk 9:119-23
- BRINK PRG, Luitse JSK, Leenen LPH. 1997. Traumatologie: verleden, heden en toekomst. Ned Tijdschr. Heelk 3:72-3.
- Andeweg CS, Vingerhoedt NM, VAN VUGT AB, Haerkens MH. 2006. Damage control surgery in polytraumatized patients. Ned Tijdschr Geneeskd 150(27):1503-7
- VAN VUGT AB, van Kampen A. 2006. An unstable pelvic ring. The killing fracture. J Bone Joint Surg Br 88(4):427-33
- Frankema SP, Steyerberg EW, Edwards MJ, VAN VUGT AB. 2005. Comparison of current injury scales for survival chance estimation: an evaluation comparing the predictive performance of the ISS, NISS, and AP scores in a Dutch local trauma registration. J Trauma 58(3):596-604
- Frankema SP, Ringburg AN, Steyerberg EW, Edwards MJ, Schipper IB, VAN VUGT AB. 2004. Beneficial effect of helicopter emergency medical services on survival of severely injured patients. Br J Surg 91(11):1520-6
- van Olden GD, VAN VUGT AB, Biert J, Goris RJ. Trauma resuscitation time. Injury. 2003 Mar;34(3):191-5
- Frankema SP, Edwards MJR, VAN VUGT AB. 2002a. Quantifying the quality of extra and intramural care on an individual basis. A pilot study. Eur J Trauma 28:295-303
- Frankema SP, Edwards MJR, Steyerberg EW, van Vugt AB. 2002b. Evaluating Quality of Care in trauma system and finding a suitable method for the Dutch situation. Eur J Trauma 2002
- VAN VUGT AB. Scientific research in trauma care; an advisory report from the Health Research Council of the Netherlands. Ned Tijdschr Geneeskd 28;146(39):1815-6

- Edwards MJR, Frankema SP, Kruit MC, Bode PJ, Breslau PJ, VAN VUGT AB. 2001. Efficiency of a standardized diagnostic protocol in trauma management. Eur J Trauma 27:81-86
- VAN VUGT AB. 2000. ['Advanced trauma life support' in Netherlands] Ned Tijdschr Geneeskd. 144(44):2093-7
- Meilink A, HEMMEN B, SEELEN H, Kwakkel G. (subm.) The effects of EMG-NMES of the extensors of the hand on the arm/hand function compared to usual care in patients with stroke. (Review)
- Spooren AIF, Snoek G, Janssen-Potten YJM, Ijzerman M, Kerckhofs E, van der Woude L, SEELEN HAM. (Subm.) Clinical outcome of Upper Extremity Skilled Performance in persons with Cervical Spinal Cord Injuries.
- Schmeets A, HEMMEN B, EVERS S, Ament A, SEELEN H. (subm) Costs and effects of a prosthesis with an electronically stance and swing phase controlled knee joint.
- HEMMEN B, SEELEN H. 2006. Effects of Movement Imagery and EMG-triggered feedback on arm-hand function in stroke patients in the sub-acute phase. Clin Rehabil (accepted)
- Spooren AIF, Janssen-Potten YJM, Post M, Kerckhofs E., Nene A, SEELEN HAM. 2006. Measuring progress in arm hand skilled performance in persons with a cervical spinal cord injury. Spinal Cord 44(12):772-9
- SEELEN H, Anemaat S, Janssen H, Deckers J. 2003. Effects of prosthesis alignment on pressure distribution at the stump/socket interface in trans-tibial amputees during unsupported stance and gait. Clin Rehabil 17:780-9
- Kilkens OJE, Post MWM, SEELEN HAM, Dallmeijer AJ, van der Woude L. 2003. Wheelchair Skills Tests, a systematic review. Clin Rehabil 17(4):418-30
- Geboers JFM, Wetzelaer W, SEELEN HAM, Spaans F, Drost MR. 2002. Ankle-foot orthosis has limited effect on walking test parameters among patients with peripheral ankle dorsiflexor paresis. J Rehabil Med 34:80-5
- van Tuijl JH, Janssen-Potten YJM, SEELEN HAM. 2002. Evaluation of upper extremity motor function in tetraplegics: a review. Spinal Cord 40:51-64
- SEELEN HAM, Potten YJM, Adam JJ., Drukker J, Spaans F, Huson A. 1998. Postural motor programming in paraplegic patients during rehabilitation. Ergonomics 41(3): 302-16
- Hofstad CJ, VAN DER LINDE H, Nienhuis B, Weerdesteyn V, Duysens J, Geurts AC. 206. High failure rates when avoiding obstacles during treadmill walking in patients with a transtibial amputation. Arch Phys Med Rehabil 87(8):1115-22
- VAN DER LINDE H, Hofstad CJ, van Limbeek J, Postema K, Geertzen JH. 2005. Use of the Delphi Technique for developing national clinical guidelines for prescription of lower-limb prostheses. J Rehabil Res Dev 42(5):693-704
- Geertzen JH, Hijmans JM, VAN DER LINDE H. 2005. Prosthetic prescription in The Netherlands. Prosthet Orthot Int 29(1):113-4
- Nawijn SE, VAN DER LINDE H, Emmelot CH, Hofstad CJ. 2005. Stump management after trans-tibial amputation: a systematic review. Prosthet Orthot Int 29(1):13-26
- VAN DER LINDE H, Hofstad CJ, Geurts AC, Postema K, Geertzen JH, van Limbeek J. 2004. A systematic literature review of the effect of different prosthetic components on human functioning with a lower-limb prosthesis. J Rehabil Res Dev 41(4):555-70
- VAN DER LINDE H, Geertzen JH, Hofstad CJ, Van Limbeek J, Postema K. 2004. Prosthetic prescription in the Netherlands: an interview with clinical experts. Prosthet Orthot Int 28(2):98-104
- EVERS S, Voss G, Nieman F, Ament A, Groot T, Lodder J, Boreas A, Blaauw G. 2002. Predicting the costs of hospital stay for stroke patients: the use of diagnoses related groups. Health Policy 61:21-42
- Struijs JN, van Genugten ML, EVERS SM, Ament AJ, Baan CA, van den Bos GA. 2006. Future costs of stroke in the Netherlands: the impact of stroke services. Int J Technol Assess Health Care 22(4):518-24
- Hendriks MR, van Haastregt JC, Diederiks JP, EVERS SM, Crebolder HF, van Eijk JT. 2005. Effectiveness and cost-effectiveness of a multidisciplinary intervention programme to prevent new falls

and functional decline among elderly persons at risk: design of a replicated randomised controlled trial [ISRCTN64716113]. BMC Public Health 14;5:6.
- EVERS SM, Struijs JN, Ament AJ, van Genugten ML, Jager JH, van den Bos GA. 2004. International comparison of stroke cost studies. Stroke 35(5):1209-15. Review.
- EVERS SM, Ament AJ, Blaauw G. 2000. Economic evaluation in stroke research : a systematic review. Stroke 31(5):1046-53. Review.
- van Mastrigt G, Maessen J, Heijmans J, SEVERENS J, Prins M. 2006. Does fast-track treatment lead to a decrease of intensive care unit and hospital length of stay in coronary artery bypass patients? Crit Care Med 34:1624-34
- Verkerk K, Van Veenendaal H, SEVERENS J, Hendriks EJ, Burgers JS. 2006. Considered judgement in evidence-based guideline development. Int J Qual Health Care 18(5):365-9

Referenties / References

- Baker et al. 1974.The Injury Severity Score: (..). J Trauma 14:187-96
- Baldry-Currens 2000. Evaluation of disability and handicap following injury. Injury 31:99-106
- Beeck van et al. 1997. Medical costs and economic production (..). J Trauma. 42(6):1116-23
- Brazier et al. (2002). "The estimation of a preference-(..)." J Health Econ 21(2): 271-92
- Brazier et al. (1992). "Validating the SF-36 (..)." BMJ 305(6846): 160-4
- Briggs et al. 1997. Pulling cost-effectiveness analysis (..). Health Econ 6(4):327-40
- Cameron et al. 2004. Co-ordinated multidisciplinary approaches for inpatient rehabilitation (..). In: The Cochrane Library, Issue 2
- Carswell et al. 2004. The Canadian Occupational Performance (..). Can J Occup Ther 71(4):210-22
- Cooper 2004. Trauma severity indices. In: Outcome measures in orthopaedics (..). Pynsent et al. (eds). London, Arnold Publ. p.70-80
- Czyrny et al. 1998. Functional outcomes of patients with multiple limb trauma Am J Phys Med Rehabil 77(5):407-11
- Davenport et al. 1996. Effect of correcting outcome data (..). BMJ 15;312(7045):1503-5
- Devilly et al. 2000. Psychometric properties of the credibility/expectancy questionnaire. J Behav Ther Exp Psychiatry 31:73-86
- Dimopoulou et al. 2004. Health-related quality of life and (..). Am J Phys Med Rehabil 83(3):171-6
- Eastwood et al. 1999. Functional status and its uses in (..). Mount Sinai Journal of Medicine 66(3):179-87
- Elliott 1999. Measuring the health outcome of general ICU patients:(..). Aust Crit Care 12(4):132-40
- Erli et al. 2000. Determinants of global quality of life (..). Chirurg 71:1132-7
- Exel van et al. 2003 Costs of stroke and stroke services: (..). http://www.resource-allocation.com/content/1/1/2
- Fern et al. 1998. Trauma patients with multiple extremity injuries (..). J Trauma 45(3):489-94
- Fernandez et al. 2001. Cognitive deficits after polytrauma. (..). Unfallchirurg 104(10):938-47
- Foa et al. 1993. Reliabilty and validity of a brief instrument (..). J Traum Stress 6(4):459-73
- Folstein et al. 1975. Mini-mental state a practical method for grading the cognitive state of patients for the clinician. J Psychiat Res 12:189–98
- Gouma et al. 2002. RGO advies onderzoek traumazorg. RGO, Den Haag
- Halcomb et al. 2005. Life beyond severe traumatic injury (..). Aust Crit Care 18(1):17-8, 20-4
- Hammacher et al. 2004. Traumatologie in perspectief: Beleidsplan Nederlandse Vereniging voor Traumatologie
- Häusler et al. 2006. Pilot study on the comprehensive economic costs of major trauma (..). J Trauma 61(3):723-31
- Herrmann 1997. International experiences with the Hospital Anxiety and Depression Scale (..). J Psychosom Res 42(1):17-41
- Hetherington et al. 1995. The disability status of injured patients measured (..). Injury 26(2):97-101
- Holtslag et al. 2007. Long-term functional health status of severely injured patients. (Injury: in press)

- Hulsebos et al. 1991. Measuring quality of life (..). Intensive Care Med 17(5):285-8
- Holbrook et al. 1998. Outcome after major trauma: Discharge and 6-month follow-up (..). J Trauma 45(2):315-24
- Holbrook et al. 1999. Outcome after major trauma: 12-month and 18-month follow-up (..). J Trauma 46(5):765-73
- Holbrook et al. 2001a. The impact of major in-hospital complications (..). J. Trauma 50:91-5
- Holbrook et al. 2001b. The importance of gender on outcome (..). J . Trauma 50:270-3
- Jackson et al. 2007. Long-term cognitive, emotional, and functional outcomes in trauma (..). J. Trauma 62(1):80–8
- Jurkovich et al. 1995. The sickness impact profile as a tool (..). J. Trauma 39:625-31
- Keijzer et al. 2000. A comparison of costs of regular and integrated (..). ISTAHC Conference, June 2000.
- Kempen et al. 2003. The role of depressive symptoms in recovery from injuries to the extremities (..). Int J Geriatr Psychiatry 18(1):14-22
- Kidd et al. 1995. The Funtional Independence Measure (..). Disabil Rehabil 17:10-4
- Kinebanian et al. 2006. Grondslagen van de Ergotherapie. Maarssen, Elsevier gezondheidszorg
- Koopmanschap 2005. PRODISQ: a modular questionnaire on productivity (..). Exp Rev Pharmacoecon Outc Res5(1):23-8
- Koopmanschap et al. 2004. Manual for the use of the PRODISQ version 2.1 (..). Rotterdam: EUR
- McColl et al. 2000. Validity and community utility of the Canadian Occupational Performance Measure. Can J Occup Ther 67(1):22-30
- Miller et al. 2000. Outcomes of trauma patients who survive prolonged lengths of stay (..). J Trauma 48(2):229-34
- Mulder et al. 2002. Setting priorities in injury prevention (..). Inj Prev 8(1):74-8
- Neugebauer et al. 2002. Quality of life after multiple trauma (..). Restor Neurol Neurosci 20(3-4):161-7
- Nirula et al. 2006. Do trauma centers improve functional outcomes (..). J Trauma 61(2):268-71
- Oostenbrink et al. 2004. Handleiding voor kostenonderzoek (..). Diemen, CvZ
- Paulus et al. 2002. ABC: The pathway to comparison of the costs (..). Public Money and Management 22(3): 25-32
- Richmond et al. 1998. A Prospective study of predictors of disability at 3 months (..). J. Trauma 44(4):635-43
- Santini et al. 2004. Complications. In: Outcome measures in orthopaedics (..). Pynsent et al. (eds). London, Arnold Publishers, p.81-94
- Sluis van der et al. 1995. Multiple injuries: an overview of the outcome. J Trauma 1995 May;38(5):681-6
- Stichting Consument en Veiligheid, 2005. Ongevallen: cijfers en kosten. http://www.veiligheid.nl
- Teasdale et al. 1974. Assessment of coma and impaired consciousness. Lancet ii, 81-84
- Trombly et al. 2002. Occupational therapy and achievement of self-identified goals (..). Am J Occup Ther 56(5):489-98
- Vles et al. (2005) Prevalence and determinants of disabilities and return (..). J Trauma 58(1):126-35
- Vree van et al. 2001. Beleidsvisie traumazorg (..). Leiden, CvZ
- Weseman 2000. Kosten van verkeersonveiligheid (..). Leidschendam, SWOV
- www.prismant.nl
- Zee van der et al. 1993. Measuring general health using the RAND-36 (..). Groningen: Noordelijk Centrum voor Gezondheidsvraagstukken
- Zelle et al. 2005a. The impact of injuries below the knee (..). Injury, Int J Care Inj 36:169-77
- Zelle et al. 2005b. Influence of workers' compensation eligibility (..). Am J Surg 190:30-6

Financiële gegevens / Financial data

DEFINITIEF

Geplande duur in maanden / Planned duration in months

36 maanden / months

ZonMw budget

	Jaar / Year								
Kostenpost / Cost item	*1*	*2*	*3*	*4*	*5*	*6*	*7*	*8*	*Totaal / Total*
Personeel	115.600	120.500	125.900	0	0	0	0	0	362.000
Materieel	0	0	0	0	0	0	0	0	0
Implementatie	0	0	0	0	0	0	0	0	0
Apparatuur	1.200	900	600	0	0	0	0	0	2.700
Overig	2.000	2.000	5.200	0	0	0	0	0	9.200
**Totaal / Total**	**118.800**	**123.400**	**131.700**	**0**	**0**	**0**	**0**	**0**	**373.900**

Co-financiering / Cofinancing

Naam co-financier / Name of cofinancier	Bedrag / Amount	Status

Bijzondere gegevens / Additional information

Vergunningen / Permits

	Vergunning nodig / Permit required?		Vergunning verkregen / Permit obtained?	
	Ja / Yes	Nee / No	Ja / Yes	Nee / No
METC/DEC	X			X
WBO		X		X
Biohazards		X		X

Andere vergunningen / Other permits

Historie subsidieaanvraag / History grant application

Deze aanvraag is eerder ingediend bij het programma /This grant application has previously been submitted to the ZonMw programme:

Projectnummer / Project number:

Ondertekening / Signatures

Naam penvoerder-projectleider:<br><br>H.A.M. Seelen	Naam bestuurlijk verantwoordelijke:<br><br>T.S. de Wit
Plaats en datum:	Plaats en datum:

DEFINITIEF

Hoensbroek, 12-2-'08	Hoensbroek, 12-2-'08
Handtekening: [signature]	Handtekening: [signature] A.M.A. Meijs

voorzitter raad van bestuur

Flow chart SFTRS study

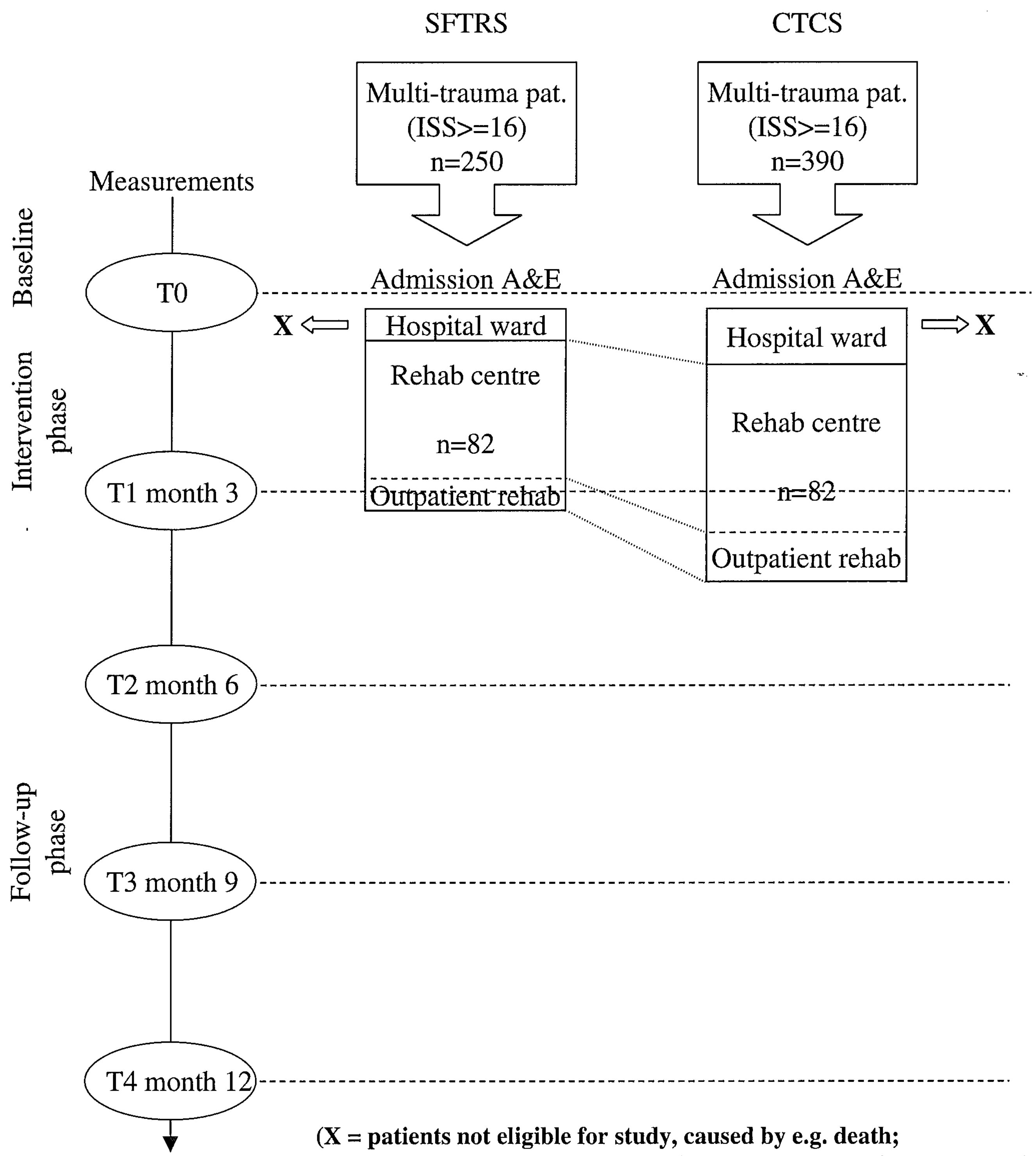

(X = patients not eligible for study, caused by e.g. death; failing to meet inclusion criteria; referred to nursing home, etc.)
